# Gene Expression, Docking and Machine Learning in Malaria Drug Discovery: A Systematic Review

**DOI:** 10.1155/bri/2724332

**Published:** 2026-02-26

**Authors:** Reuben Samson Dangana, Israel Ehizuelen Ebhohimen, Samson Anjikwi Malgwi, Samuel Chima Ugbaja, Moses Okpeku

**Affiliations:** ^1^ Discipline of Genetics, School of Life Sciences, University of KwaZulu-Natal (Westville), Durban, South Africa; ^2^ Department of Biochemistry, Faculty of Life Sciences, Ambrose Alli University, PMB 14, Ekpoma, Nigeria, aauekpoma.edu.ng; ^3^ Discipline of Traditional Medicine, School of Medicine, University of KwaZulu-Natal, Durban, South Africa, ukzn.ac.za

**Keywords:** Antimalarial drug discovery, gene expression profiling, machine learning, molecular docking, *Plasmodium* species

## Abstract

**Background:**

Malaria remains a significant and worldwide health threat with increasing resistance to current treatments, stimulating the demand for innovative approaches in pursuing drug discovery. This systematic review integrates the progress made from 2014 through 2024 regarding molecular methods like gene expression profiling, molecular docking and machine learning to understand the biology of *Plasmodium* and identify new drug targets and compounds, focusing on herbal remedies and computational methods.

**Methodology:**

Several studies were found using a PRISMA‐guided search of PubMed, Scopus and Web of Science (64 studies found). The data extracted were gene expression outcomes, docking affinities, ML models and experimental validations (in vitro/in vivo).

**Results:**

Molecular docking emerged as the dominant technique (32.37%), followed by in vitro antiplasmodial assays (14.39%), ADMET profiling (10.79%) and gene expression studies (3.60%). RNA‐seq analysis revealed key host and parasite genes modulated by herbal treatments, including those involved in apoptosis and inflammation. Notably, compounds like isorhamnetin and myricetin 3‐O‐glucoside showed exceptionally high binding affinities to *Plasmepsin II* and *Plasmodium falciparum* lactate dehydrogenase (PfLDH) (ΔG < −13 kcal/mol). ML models like random forest and support vector machine (SVM) exhibited high predictive results (AUC value up to 0.87) for bioactivity and resistance patterns that showed flavonoids (quercetin) and terpenoids (eugenol) as good candidates. Pathways that are often attacked are haemoglobin degradation, glycolysis, pyrimidine metabolism and protein synthesis.

**Conclusion:**

Multiomics, docking and ML integration improve the target identification and prioritise the compounds. This review illustrates the great potential of molecular techniques for the development of drugs against antimalarial helicases that are not resistant to drug therapy. However, in vivo data holes and methodology inconsistency limit clinical translation. Future work should include standardisation of protocols and studies of synergistic combinations of phytochemicals.

**Highlights of the Study**
• Molecular docking identified nonstandard antimalarials like isorhamnetin (−156.33 kcal/mol, *Plasmepsin II*), targeting haemoglobin degradation.
• RNA‐seq showed herbal extracts modulating *Plasmodium* genes (PfGEXPS) and host immunity (*IL-6*), disrupting parasite development.
• ML models (KNN, Chemprop) predicted phytochemical bioactivity with AUC 0.87–0.95, enhancing antimalarial screening.
• Multiomics and network pharmacology prioritised *PfDHODH*, integrating docking and proteomics to combat resistance.
• Techniques targeted *Plasmodium* pathways like glycolysis and folate metabolism, validated in vitro, supporting multistage therapies.

## 1. Introduction

Malaria remains one of the most pressing global health challenges, with significant socioeconomic impacts, particularly in low‐ and middle‐income countries [1, 2]. According to the World Health Organisation (WHO), an estimated 247 million cases of malaria and 619,000 related deaths occurred in 2021, with the vast majority reported in sub‐Saharan Africa. In 2022, malaria disease increased to 249 million cases in 85 countries, most of which were in Africa (93.6%), the Eastern Mediterranean (3.3%), Southeast Asia (2.1%), the Western Pacific (0.8%) and the Americas (0.2%) [3–6]. The child population aged below 5 years contributes to the deaths by almost 79%, and this shows the incessant vulnerability of the population [2]. The deadliest of the malaria‐causing parasites, *Plasmodium falciparum*, has evolved resistance to almost all first‐line antimalarial treatments over time [7], including the artemisinin‐based combination therapy (ACT), which is now regarded as the gold standard in malaria treatment. Drug resistance is a current menace that highlights the importance of novel research methods in enhancing malaria control and treatment interventions.

Over the past few years, molecular technologies have turned out to be an invaluable asset in malaria research, helping scientists to unravel the complicated biology of *Plasmodium* species and providing a way to speed up drug discovery. Among them, one can distinguish gene expression profiling, molecular docking and machine learning (ML) because of their influence and relevance to different fields of research. RNA sequencing (RNA‐seq), especially at the single‐cell scale, has also been used to offer information on the biology of parasites by showing cell–cell variability when it comes to gene expression [8]. This degree of granularity is essential to the comprehension of stage‐specific transcriptional programmes that cannot be identified by bulk transcriptomics. As an example, scRNA‐seq has been applied to dissect the life cycle of *Plasmodium* and has helped identify genes that mediate the switch between asexual and sexual stages that are critical to its transmission and survival [2, 9]. Another application of RNA‐seq is the evaluation of the changes in gene expression with respect to environmental signals and drug exposure, such as herbal intervention, though this field is not thoroughly studied yet [10].

Molecular docking is used as an adjunct method in that researchers can predict how the small molecules interact with *Plasmodium* proteins. This is a critical computational approach to determine binding affinities and possible drug‐target interactions and therefore inform experimental validation and rational drug design [11]. The power of docking studies is specifically strong in conjunction with the omics data, and it can be used to explore the protein function and ligand specificity in a more systematic way. The last studies have used deep learning docking methods, that is, the diffusion model (Targetdiff), to generate and screen novel compounds using structural features of essential Plasmodium proteins [2, 9, 12].

Another transformative tool is ML, which has made breakthroughs in the analysis of large‐scale biological data in malaria studies. ML algorithms are effective in the classification of compounds as active or inactive, drug resistance pattern prediction, and the optimal selection of lead compounds to be subjected to experimental testing, based on molecular descriptors [13]. Various experiments have indicated that the random forest (RF), support vector machines (SVMs) and artificial neural network models can be used to predict antimalarial compounds with a high level of accuracy [9, 12, 14, 15]. Notably, feature selection methods help in boosting the performance of these models by selecting the most informative descriptors, thereby improving the performance of the models at the expense of low computational complexity.

The systematic review examines the role of the modern state‐of‐the‐art molecular and computational tools in revolutionising malaria research, and three areas are discussed as follows. First, it discusses the capability of RNA‐seq to trace genetic changes in *Plasmodium* parasites that have encountered natural therapies, which sheds some light on the way in which traditional therapies can interfere with the process of infections. Second, it also measures the accuracy of molecular docking to study drug‐target interactions and thus its importance in the search for promising compounds among large chemical libraries. Lastly, it evaluates the twofold influence of ML in forecasting the emergent drug resistance trends as well as simplifying the process of identifying new antimalarial compounds. The review highlights the potential of combining laboratory‐based molecular understanding with computational creativity to speed up advances in one of the oldest infectious diseases that has reached humankind by exploring the extent to which these tools can be effective in overcoming critical difficulties, such as treatment modification and bottlenecks in drug development.

## 2. Methods

### 2.1. Database Search Process

The Preferred Reporting Items of Systematic Reviews and Meta‐Analyses (PRISMA) guidelines [16] were used to complete this systematic review. Three major electronic databases (PubMed, Scopus and Web of Science) were searched. The search was conducted to find the studies that were applicable to the analysis of applying molecular techniques in malaria research, especially the studies that involved gene expression profiling, molecular docking and ML. A combination of the following search terms was used: (Plasmodium OR malaria) AND (RNA sequencing OR gene expression) AND (herbal treatment), as well as (machine learning OR AI) AND (drug resistance prediction). Peer‐reviewed articles published in English were considered, published between the year 2014 and 2024.

### 2.2. Criteria for Eligibility

Studies were also included in case they satisfied several predetermined criteria. Only studies that used gene expression profiling, molecular docking or machine learning methods were included in the scope of malaria research. Only the studies that are related to the *Plasmodium* species, including *P. falciparum*, *P. vivax* or *P. berghei,* were taken into consideration. The research that involved the use of natural products or herbal plants as an intervention was taken into consideration. To be eligible, the studies had to be primary research articles, and they needed to have computational or in silico analyses and have been published in English in the last 10 years, 2014–2024, in any peer‐reviewed journal. In addition, the inclusion criteria required that the molecular procedure should be well outlined and its use in malaria studies should be explicitly stated. Articles were filtered by eliminating those that were not molecular, malaria‐unrelated, non‐*Plasmodium*‐related, opinion pieces, editors, conference abstracts, reviews or preprints that were not peer reviewed. Articles that were older than the specified range of dates of publications or were not in English and articles that were not clear on the method or technique used were also excluded.

### 2.3. Data Extraction

The systematic selection of data on eligible articles by using a predesigned template, specific to the objectives of the review, was used to collect crucial information related to gene expression profiling, molecular docking and ML studies on malaria. Fields that were extracted were as follows: study title, year of publication, study design/type, species/strain of *Plasmodium*, sample size, herbal treatment/intervention, comparator group/standard, technique used, key genes (RNA‐seq), affected pathways, validation method, docking software, target protein (PDB ID), ligands screened, binding affinity (ΔG), half maximal inhibitory concentration (IC_50_), ML model, input data type, prediction output, AUC/accuracy, limitations, key findings, research gaps and reference/author. Data were extracted by two reviewers into an Excel spreadsheet, and differences were resolved by agreement. Missing data were denoted as ‘Not Reported’, and supporting information was used where possible so that a complete and reproducible synthesis of findings was done [17].

### 2.4. Quality Assessment

The quality of included studies had been assessed with a stringent level so that the reliability and validity of the findings could be considered credible in this systematic review. Articles found in e‐libraries were uploaded to Rayyan [18], a web‐based application, to undergo independent screening. The scientific names of plants used in the studies were picked and cross‐tabulated to ascertain accuracy in the scientific name using the World Flora Online (https://wfoplantlist.org) to ensure taxonomic accuracy. Study integrity assessment criteria were specific to the gene expression profiling, molecular docking and ML articles, which were implemented to test, study design, methodology and reporting to guarantee a strong evidence synthesis of the molecular techniques in malaria research.

## 3. Results

### 3.1. Overview of Included Studies

**FIGURE 1:**
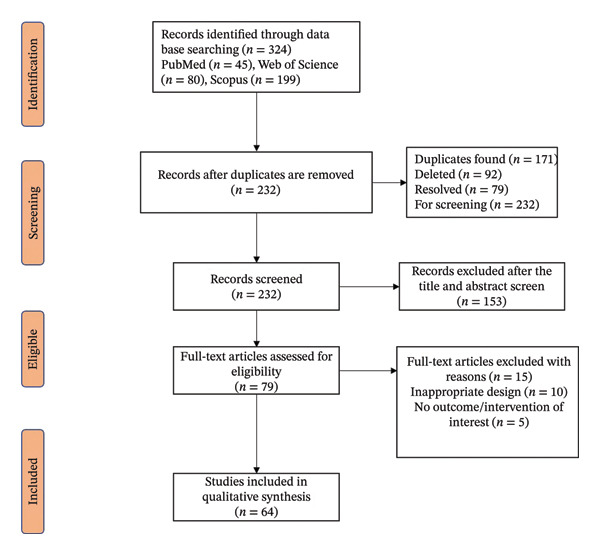
Prisma flowchart for study selection.

The systematic review identified 324 records through database searches (PubMed: 45, Web of Science: 80, Scopus: 199), with 232 unique records after duplicate removal. Following title and abstract screening, 153 records were excluded, and 79 full‐text articles were assessed for eligibility. Fifteen articles were excluded due to inappropriate design (*n* = 10) or lack of relevant outcomes/interventions (*n* = 5), resulting in 64 studies included in the qualitative synthesis; see Figure 1. Among 64 studies analysed for molecular techniques, molecular docking was identified as the most commonly employed method, constituting 32.37% of the 139 total technique findings. In vitro antiplasmodial assays were utilised in 14.39% of the findings, while ADMET profiling was applied in 10.79% of cases. Cytotoxicity assays were documented in 9.35% of the findings, and GC–MS was used in 8.63%. NMR spectroscopy was employed in 7.19% of cases, followed by molecular dynamics (MDs) simulation at 6.47%. Bioassay‐guided fractionation was reported in 5.04% of the findings, and gene expression techniques were noted in 3.60%. ML and UPLC–QTOF–MS were each recorded in 2.16% of the findings, while 3D homology modelling, MM/GBSA or MM/PBSA analysis, 3D molecular modelling and energy minimisation were each implemented in 1.44% of cases. These results highlight the predominant role of docking and profiling methods in advancing molecular research on malaria. Table 1 also provides a comprehensive review of methodologies in antimalarial drug discovery: in silico, in vitro, in vivo, chemical characterisation, docking and validation strategies reported by included studies.

**TABLE 1:** Comprehensive review of methodologies in antimalarial drug discovery*:* in silico, in vitro, in vivo, chemical characterisation, docking and validation strategies.

References	In silico methods	In vitro methods	In vivo methods	Chemical analysis and compound characterisation	Docking software	Validation method
Adelusi et al. [19]	3D homology modelling (I‐TASSER, Rosettafold), ADMET profiling (ADMETlab 2.0), molecular docking, molecular dynamics (GROMACS), MM/GBSA analysis	—	—	—	AutoDock Vina v1.5.7	Ramachandran plot, ProSA‐web z‐score, RMSD comparison of I‐TASSER and Rosettafold models
Onguéné et al. [20]	3D molecular modelling (MOE, LigPrep), ADMET descriptor calculation (QikProp), energy minimisation (MMFF94, OPLS force fields)	—	—	—	—	Comparison with Lipinski’s Rule of Five, Jorgensen’s Rule of Three, ADMET descriptors of known drugs
Happi et al. [21]	Molecular docking, ADMET prediction (ADMETLab 2.0)	Bioassay‐guided fractionation, antiplasmodial assay (schizont maturation inhibition), cytotoxicity assay (MTT on P388 cells)	—	—	PyRx/Vina, BIOVIA Discovery Studio	IC50 values, selectivity index (SI = Cytotoxicity IC50/Antiplasmodial IC50), docking binding energies (ΔG)
Zininga, et al. [22]	—	Chaperone activity assay (MDH aggregation), ATPase activity assay, antiplasmodial assay (pLDH), protein expression/purification (PfHsp70‐1, PfHsp70‐z)	—	UPLC–QTOF–MS (phenolic quantification)	—	Chaperone assay normalised to spontaneous MDH aggregation, ATPase assay normalised to basal activity, pLDH assay validated with chloroquine (IC_50_: 8.5 ng/mL), SDS–PAGE for protein purity
Khan et al. [23]	Molecular docking	DPPH radical scavenging, reducing power assay, total antioxidant capacity, pLDH assay	—	—	MOE	IC50 (antiplasmodial), EC50 (antioxidant), Moldock score, pKi
Elmaidomy et al. [24]	PASS virtual screening, molecular docking, MDS (Desmond, 50 ns), free energy perturbation (FEP)	Malstat assay (*P. falciparum* growth inhibition)	—	1H, DEPT‐Q, HSQC, HMBC NMR; HRESIMS; UV; IR	AutoDock/Vina	PASS prediction (Pa: 0.892 for cytochrome‐C reductase inhibition), docking/MDS binding to cytochrome bcl (ΔG: −8.33 kcal/mol vs. −14.46 kcal/mol for atovaquone)
Knockleby et al. [25]	Molecular docking	Cytotoxicity (SRB), anti‐plasmodial (HRP2 ELISA), cell cycle (flow cytometry), apoptosis (Annexin V), protein analysis (Western blot), kinase assay (ADP‐Glo)	—	NMR, HR‐ESIMS	PyRx (0.9.8) with Autodock 4.2	Spectroscopic comparison with standards (NMR, MS), repeated experiments (*n* ≥ 2 for cytotoxicity, *n* = 5 for antiplasmodial), 1‐way ANOVA for apoptosis, nonlinear regression for IC50
Gomes et al. [26]	Molecular docking	Oxidative stress (TEAC, GSH, TBARS)	Antimalarial activity (4‐day suppressive test)	NMR	Molegro Virtual Docker 5.5	Compound identification via NMR, repeated oxidative stress measurements, statistical significance (*p* ≤ 0.05), MolDock rerank scores
Rawa et al. [27]	Molecular docking, MD simulations (AMBER 18), MM–PBSA, computational NMR, ECD (DFT, TD‐DFT)	Antimalarial (*P. falciparum* growth via LDH)	—	HR‐ESI‐TOF‐MS, HR‐EI‐TOF‐MS, NMR, DEPT, HSQC, HMBC, DQF‐COSY, NOESY	AutoDock 4.2	Redocking of co‐crystallised ligand (RMSD ∼2.0 Å), MD stability (RMSD, RMSF, Rg, H‐bond profiles), MM–PBSA, ECD spectra comparison
Al‐Huqail et al. [28]	Molecular docking	IC_50_ via sigmoidal dose–response curves	Parasitaemia, suppression, survival time	UPLC–ESI–MS/MS (20 compounds), HPLC (rutin: 3.65 mg/g, hesperidin: 36.17 mg/g)	Accelrys Discovery Studio 2.5	HPLC accuracy (99.70%–100.23%), precision (%RSD 1.38–3.07), LOD/LOQ, docking on PfDHFR–TS (1J3I), Pf‐PMT (2UJ9), Lm‐PTR1 (2BFM), Lm‐FPPS (4JZX)
Ikpa et al. [29]	Molecular docking, ADMET (admetSAR), drug likeness (OSIRIS), toxicity risk assessment	—	—	FTIR, GC–MS	—	FTIR, GC–MS characterisation, OSIRIS toxicity/drug likeness, admetSAR ADMET, docking binding affinity, 2D/3D interaction visualisation
Enyiekere et al. [30]	Molecular docking, ADMET profiling	Antiplasmodial assays (suppressive, prophylactic, schizonticidal)	Parasitaemia reduction, chemosuppression, survival time	GC–MS	PyRx, BIOVIA Discovery Studio	Comparison with chloroquine/pyrimethamine, docking/ADMET profiling
Olaosebikan et al. [31]	Molecular docking, ADMET prediction	—	—	GC–MS	AutoDock Vina	Redocking the native ligand (DSM1, RMSD 0.15 Å), ADMETlab 2.0 predictions
Pradhan et al. [32]	Molecular docking	In vitro antimalarial (modified WHO MARKIII)	—	GC–MS	Biovia Discovery Studio 2021	Dose–response curves, microscopic parasite clearance
Wiraswati et al. [33]	Molecular docking	Antiplasmodial (Giemsa staining, microscopy)	—	GC–MS	PyRx, AutoDock Vina	—
Herrera‐Calderon et al. [34]	Molecular docking, MD (Desmond 2020.1)	Antioxidant assays (DPPH, ABTS, FRAP)	—	GC–MS	AutoDock 4.2	RMSD, RMSF, Rg, SASA
Kane et al. [35]	Molecular docking	Gene expression (qPCR)	—	GC–MS	AutoDock Vina (via PyRx)	qPCR for gene expression, GC–MS compound identification, docking binding affinity
Deligianni et al. [15]	—	High‐content microscopy (GFP‐tagged parasites)	—	GC–MS	—	Inhibition > 50% (*p* ≤ 0.01), ookinete conversion rate inhibition > 50%, in vitro testing of ML‐predicted compounds
Samuel et al. [36]	High‐throughput virtual screening, molecular docking, pharmacophore modelling, ADME, toxicity profiling	—	—	—	—	Binding affinity scores, molecular interaction analysis (H‐bonds, hydrophobic), pharmacophore modelling, ADMET predictions
Shekari et al. [37]	Homology modelling, molecular docking, ADME–Tox, PCA, energy minimisation, Ramachandran plot	—	—	—	—	Homology model quality (GMQE, sequence identity, Ramachandran plot), binding affinity, molecular interactions (H‐bonds, pi‐alkyl, pi‐cation/anion), ADME–Tox (SwissADME, OSIRIS), PCA correlations
Adeoye et al. [38]	Molecular docking	In vitro β‐haematin inhibition	—	HPLC–DAD, HRGC–MS	AutoDock 4.2, AutoDock Vina	—
Taranto et al. [39]	Molecular docking, comparative homology modelling	—	—	HPLC–MS/MS, NMR	AutoDock Vina	—
Chaniad et al. [40]	Molecular docking	Hypoxanthine uptake inhibition, MTT (cytotoxicity)	—	—	AutoDock 4.2, UCSF Chimaera 1.14, PLIP	^3^H‐hypoxanthine uptake, MTT assay, docking binding interactions
Birgit Viira [41]	SVM classification, molecular descriptors (ISIDA), virtual screening	Antiplasmodial (SYBR Green I), cytotoxicity (resazurin, MRC‐5)	—	—	—	Cross validation (leave‐1/3‐out, balanced accuracy > 0.7), consensus voting (> 70% active likelihood), IC_50_ via ICEstimator, selectivity index (CC_50_/IC_50_)
Rehman [42]	Molecular docking, binding pocket prediction (CASTp), visualisation (PyMOL, LigPlot+), network pharmacology, pathway enrichment	Isothermal Titration Calorimetry (ITC)	—	—	AutoDock Vina 4.2, AutoDock Tools 1.5.7	Binding energy (ΔG), STRING DB PPI (*p* value: 6.86*e* − 09), pathway enrichment (FDR < 0.001, strength > 0.5), ITC (Ka, ΔH, *n*), spectrophotometric protein/ligand concentrations
Konyanee et al. [43]	Molecular docking, ADMET (SwissADME, ProTox‐II)	Antiplasmodial (pLDH), cytotoxicity (MTT, Vero cells)	—	NMR spectroscopy	AutoDock Vina v. 1.1.2, PyMOL v. 2.5.2, PLIP	IC_50_ determination, CC_50_ and selectivity index (SI), redocking NADH (RMSD = 0.775 Å), NMR/literature comparison
Camara et al. [44]	—	Antiplasmodial (SYBR Green I), anti‐inflammatory (RT‐qPCR, ELISA), antioxidant (LUCS), brain cell analysis (flow cytometry), gene expression (RT‐qPCR), cytokine levels (ELISA), acute oral toxicity	ECM model (parasitaemia, survival, RMCBS)	UHPLC–HRMS (MzMine, MSDIAL, MS‐FINDER)	—	IC_50_ via nonlinear regression, LDH assay, EC_50_ for antioxidant index, parasitaemia suppression, survival curves, RMCBS, GAPDH as qPCR control, HRMS/MS fragmentation vs. DNP database
Asanga et al. [45]	Molecular docking, ADME (SwissADME)	—	Antiplasmodial (Rane’s curative test)	GC–MS	AutoDock Vina 4.2, Biovia Discovery Studio	Parasite density, % growth inhibition, GC–MS characterisation, docking protein–ligand interactions, ADME analysis
Apeh et al. [46]	Molecular docking, ADMET (SwissADME)	—	4‐day suppressive test, haematological analysis	—	PyRx v0.8, AutoDock Vina	Binding affinity, interaction analysis, parasitaemia suppression, haematological indices
Nwonuma et al. [47]	Molecular docking, molecular dynamics (100 ns)	—	Parasitaemia, biochemical assays (lipid, enzyme), histopathology	—	PyRx 0.8, GROMACS	Binding affinity, MD stability (RMSD, RMSF, Rg), parasitaemia reduction, biochemical parameter analysis
Chaurasia and Pandey [48]	Molecular docking, MD simulation (100 ns), SWISSADME	β‐Haematin assay	—	GC–MS	AutoDock	Comparison with artemisinin (binding affinities, β‐haematin efficiency: 91.7%), RMSD, RMSF, Rg, SASA, Lipinski’s rule, SWISSADME, GC–MS retention times/mass spectra
Soeiro et al. [49]	Molecular docking, NMR structural analysis	Antiplasmodial, cytotoxicity (Vero cells)	Parasitaemia clearance (*P. berghei*)	Molecular modelling (drug interactions)	—	IC_50_ vs. chloroquine, parasitaemia clearance vs. chloroquine, NMR (PfHMGB1, GLR‐HsHMGB1), glabridin cytotoxicity (SI = 9.6), docking interaction energies (ΔE, ΔG)
Dzouemo et al. [50]	Molecular docking, ADMET predictions	Antiplasmodial (SYBR Green I)	—	NMR (1H, 13C), EI‐MS	AutoDock Vina (Version 1.2.0)	Spectroscopic data vs. literature, antiplasmodial assay vs. chloroquine/artemisinin, BIOVIA Discovery Studio visualisation
Samuel [51]	Molecular docking, ADMET predictions	β‐Haematin inhibition	—	NMR (1H, 13C), FTIR	AutoDock Vina (Vina Dock Wizard)	Spectroscopic data vs. literature, β‐haematin assay vs. chloroquine/artesunate, redocking of the *pfENR* ligand
Hidayati et al. [52]	Molecular docking	Antimalarial (Giemsa‐stained microscopy)	—	NMR, MS spectra, TLC	Molegro Virtual Docker 5.5	—
Adams et al. [53]	Molecular docking, MD simulation, MM–PBSA	—	—	LC‐ESI‐Q‐TOF‐MS	AutoDock Vina v.1.2.0, PyRx 0.8	ROC curve (AUC = 0.733), RMSD of redocked ligand (0.959 Å)
Sikam et al. [54]	Molecular docking, ADMET studies	In vitro antiplasmodial (schizont maturation inhibition)	—	LC‐ESI‐Q‐TOF‐MS, NMR	AutoDock Vina®	IC_50_ vs. chloroquine
Kadioglu et al. [55]	Molecular docking	Cytotoxicity (AC16), microarray, zebrafish toxicity	—	—	AutoDock 4, AutoDock Tools 1.5.7	—
Muema et al. [56]	Molecular docking, ADMET (SWISSADME)	SYBR Green I fluorescence, IEV susceptibility, gametocyte morphology	—	—	AutoDock Vina, PyRx	In vitro and ex vivo susceptibility assays
Elmaidomy et al. [57]	Inverse docking, molecular docking, MD simulation (Desmond), PPI network (Cytoscape)	Malstat assay (pLDH)	—	NMR (1H, 13C, DEPT‐Q), HRESIMS	AutoDock Vina	Malstat assay validation, Cytoscape PPI network
Snider and Weathers [58]	—	Microscopy (Giemsa), RT‐qPCR (gametocyte genes)	—	GC–MS (artemisinin quantification)	—	Microscopic quantification of parasitaemia/gametocytaemia, RT‐qPCR for gene expression changes
Tjitraresmi et al. [59]	Molecular docking, pharmacokinetic/toxicity (PreADMET, Toxtree), MD (AMBER 16), Lipinski’s Rule	—	—	—	AutoDock 4.2.6	Redocking chloroquine (RMSD = 1.13 Å), MD stability (RMSD, RMSF)
Singh et al. [60]	Molecular docking	Antimalarial (Giemsa), hemocompatibility, MTT (HEK293)	—	UV–Vis, DLS, Zeta Potential, FTIR, XRD, FESEM	AutoDock (blind docking), Schrödinger (Glide, Sitemap)	Docking scores, glide energy, parasitaemia reduction, IC_50_, hemocompatibility, MTT, physicochemical characterisation (UV–Vis, DLS, Zeta, FTIR, XRD, FESEM)
Murugesan and Kaleeswaran [61]	Molecular docking, ADME/Tox (SWISS‐ADME), ProtParam	—	—	—	AutoDock Vina, PyRx	PROCHECK, ERRAT for protein structure, PASS server for toxicity

*Note:* STRING DB, Search Tool for the Retrieval of Interacting Genes/Proteins; HRP2 ELISA, histidine‐rich protein 2 enzyme‐linked immunosorbent assay; PfLDH, *Plasmodium falciparum* lactate dehydrogenase; Plasmepsin II, aspartic protease involved in haemoglobin degradation; pLDH, parasite lactate dehydrogenase; PK/PD, pharmacokinetics/pharmacodynamics; Lipinski’s Rule, Rule of Five (drug‐likeness criteria); TBARS, thiobarbituric acid reactive substances.

Abbreviations: ADMET, absorption, distribution, metabolism, excretion, toxicity; ECM, experimental cerebral malaria; GMQE, global model quality estimate; MM/GBSA, molecular mechanics/generalized Born surface area; PASS, prediction of activity spectra for substances; RMCBS, Rapid Murine Coma and Behaviour Scale; TEAC, Trolox equivalent antioxidant capacity; UPLC–QTOF–MS, ultra‐performance liquid chromatography–quadrupole time‐of‐flight mass spectrometry.

### 3.2. Gene Expression Profiling

The application of RNA‐seq to investigate *Plasmodium* gene expression has been extensively explored across the included studies. Table 2 gives a summary of key genes grouped by affected pathways in malaria, with functional descriptions, with notable findings related to gene expression changes induced by herbal treatments. In a study by Dkhil et al. [16], female mice upregulation of *Ccl8, Saa3, Cd209a* and *Cd209b* was observed in response to *Indigofera oblongifolia* leaf extract (IOLE), while 24 immune response genes were downregulated, indicating a complex immunomodulatory effect. Similarly, Dkhil et al. [62] reported that IOLE modulated the expression of apoptotic genes (*Bcl2, Bax, Caspase-3*) and cytokine genes (*IL-1β, IL-6, IFN-γ, TNF-α*), suggesting its potential to influence both programmed cell death and inflammatory pathways during malaria infection. Another investigation by Kadioglu et al. [55] identified upregulation of *APP, CCDD1, NT5E, PCNA, PRNP, STK39* and *TXNIP* in cells of zebrafish larvae treated with artemisinin derivatives, such as artemisinin B and deoxydihydro‐artemisinin, highlighting their role in cellular proliferation and stress response pathways. Additionally, Miao et al. [14] demonstrated, via in silico studies, that *Cordia myxa* compounds altered the expression of IL‐6, CASP3, PTGS2, SRC, HMOX1, MMP‐9, APOE, NOS3, EGFR and MMP‐2, thereby affecting inflammatory, oxidative stress and extracellular matrix remodelling pathways. Camara et al. [44] reported in *P. berghei*‐infected mice modulation of proinflammatory (*IL-1β, TNF, IL-12, IFN-γ, CD11b, TLR2, NFkB, PGES, iNOS*), anti‐inflammatory (*TGFβ, CD36, HO-1*), endothelial activation (*ICAM-1, VEGF*) and cytotoxic (Granzyme B) genes in response to methanolic crude extract of *Terminalia albida* stem bark, with *GAPDH* and *P. berghei*‐specific primers used as controls. Snider and Weathers [58] observed altered expression of gametocyte markers *PfGEXPS* (early‐stage) and *Pfs25* (late‐stage) in response to *Artemisia annua* and *Artemisia afra* tea infusions from NF54 *P. falciparum* asexual parasites, indicating potential interference with parasite sexual development.

**TABLE 2:** Key genes grouped by affected pathways in malaria with functional descriptions.

Affected pathway	Pathway description	Key gene (RNA seq)	Specific gene function	References
Apoptosis	Programmed cell death eliminates infected cells or regulates immune responses in malaria.	Bax	Pro‐apoptotic protein; promotes mitochondrial membrane permeabilisation	[62]
		Bcl2	Antiapoptotic protein; inhibits programmed cell death by regulating mitochondrial membrane	[62]
		Caspase‐3	Executioner caspase cleaves cellular proteins to execute apoptosis	[62]

Inflammation/immune response	Immune activation to combat *Plasmodium* infection, potentially causing tissue damage if excessive.	CD11b	Integrin mediates leucocyte adhesion and migration in immune response	[44]
		IFN‐I^3^	Cytokine: activates macrophages, enhances antigen presentation	[44, 62]
		IL‐1I^2^	Proinflammatory cytokine; activates immune cells and induces fever	[44, 62]
		IL‐6	Cytokine regulates immune response, inflammation and haematopoiesis	[62]
		IL‐12	Cytokine stimulates T‐cell and NK‐cell activity, enhances IFN‐Î^3^ production	[44]
		iNOS	Inducible nitric oxide synthase, produces nitric oxide, enhances pathogen killing	[44]
		NFkB	Transcription factor; regulates inflammatory and immune gene expression	[44]
		PGES	Prostaglandin E synthase; produces prostaglandins, amplifies inflammation	[44]
		TLR2	Toll‐like receptor; recognises pathogens, initiates inflammatory signalling	[44]
		TNF	Proinflammatory cytokine; promotes inflammation and cytotoxicity in infected cells	[44]
		TNF‐I	Proinflammatory cytokine; induces inflammation and apoptosis in infected cells	[62]

Immune signalling/activation (upregulated)	Enhanced immune cell recruitment and pathogen recognition in response to malaria infection.	Ccl8	Chemokine; attracts monocytes and lymphocytes, upregulated in immune response	[63]
		Cd209a	C‐type lectin receptor; mediates pathogen recognition, upregulated in immune response	[63]
		Cd209b	C‐type lectin receptor; involved in immune cell adhesion, upregulated	[63]
		Saa3	Serum amyloid A3, an acute‐phase protein, promotes inflammation (upregulated)	[63]

Cellular stress and related processes	Stress responses, cell proliferation or immune modulation induced by antimalarial compounds like artemisinin B.	APP	Amyloid precursor protein; involved in neuronal signalling, upregulated in artemisinin B‐treated cells	[55]
		CCDD1	Unknown function; likely involved in cellular stress response (upregulated by artemisinin B)	[55]
		NT5E	Ecto‐5′‐nucleotidase; hydrolyses extracellular nucleotides, modulates immune response	[55]
		PCNA	Proliferating cell nuclear antigen; facilitates DNA replication and repair	[55]
		PRNP	Prion protein; involved in cellular signalling, upregulated in stress response	[55]
		STK39	Serine/threonine kinase; regulates cellular stress and ion transport	[55]
		TXNIP	Thioredoxin‐interacting protein; inhibits antioxidant pathways, promotes oxidative stress	[55]

Cell signalling, antimalarial activity	Signalling pathways targeted to disrupt *Plasmodium* survival or enhance antimalarial effects.	EphA2	Receptor tyrosine kinase; mediates cell–cell signalling, a potential antimalarial target	[64]
		ephrin‐A	Ligand for EphA2; facilitates cell signalling in immune and endothelial cells	[64]

Fatty acid biosynthesis (*Plasmodium*‐specific)	Essential for *Plasmodium* membrane synthesis, a key target for antimalarial drugs.	Fab_I (ENR)	Enoyl‐ACP reductase; catalyses fatty acid elongation in *Plasmodium*	[35]
		Fab_Z	β‐hydroxyacyl‐ACP dehydratase; catalyses dehydration in *Plasmodium* fatty acid synthesis	[35]

Housekeeping (cellular structure, metabolism)	Baseline cellular are functions used as a reference in gene expression studies.	Actin	Cytoskeletal protein; maintains cell structure, used as a housekeeping gene	[35]
		GAPDH	Glyceraldehyde‐3‐phosphate dehydrogenase; catalyses glycolysis, a housekeeping gene	[44]

Inflammation, apoptosis, oxidative stress	Multifaceted host response to malaria, balancing inflammation, cell death and oxidative stress.	APOE	Apolipoprotein E regulates lipid metabolism, modulates inflammation	[14]
		CASP3	Executioner caspase activates apoptosis by cleaving cellular substrates	[14]
		EGFR	Epidermal growth factor receptor promotes cell proliferation and survival	[14]
		HMOX1	Heme oxygenase‐1; degrades heme, reduces oxidative stress	[14]
		IL6	Cytokine promotes inflammation and immune cell differentiation	[14]
		MMP2	Matrix metalloproteinase‐2; degrades extracellular matrix, involved in tissue remodelling	[14]
		MMP9	Matrix metalloproteinase‐9; degrades extracellular matrix, aids immune cell migration	[14]
		NOS3	Endothelial nitric oxide synthase; produces nitric oxide, regulates vascular tone	[14]
		PTGS2	Cyclooxygenase‐2; synthesises prostaglandins, promotes inflammation	[14]
		SRC	Nonreceptor tyrosine kinase; regulates cell signalling and survival	[14]

Malaria parasite development (early gametocyte)	Early gametocyte formation in *Plasmodium*, critical for parasite lifecycle progression.	PfGEXPS	Exported protein; marker for early‐stage gametocyte development in *Plasmodium*	[58]

Malaria parasite development (late gametocyte)	The late gametocyte stage in *Plasmodium*, essential for transmission to mosquitoes.	Pfs25	Surface protein; marker for late‐stage gametocyte transmission in *Plasmodium*	[58]

Anti‐inflammatory response	Suppression of excessive inflammation to limit host tissue damage in malaria.	CD36	Scavenger receptor; binds oxidised LDL, modulates immune response	[44]
		HO‐1	Heme oxygenase‐1; degrades heme, exerts anti‐inflammatory effects	[44]
		TGF‐β	Cytokine; suppresses inflammation, promotes immune tolerance	[44]

Endothelial activation	Vascular changes facilitating immune cell adhesion and response in malaria infection.	ICAM‐1	Intercellular adhesion molecule‐1 facilitates leucocyte‐endothelial interactions	[44]
		VEGF	Vascular endothelial growth factor promotes angiogenesis and vascular permeability	[44]

Cytotoxicity	Direct killing of infected cells by immune effectors during the malaria immune response.	Granzyme B	Serine protease induces apoptosis in target cells during cytotoxic response	[44]

Malaria parasite detection	Molecular detection of *Plasmodium* infection via RT‐qPCR for diagnostic purposes.	*P. berghei*‐specific primers	Unknown specific genes; used for detecting *P. berghei* infection in RT‐qPCR	[44]

Techniques such as single‐cell RNA‐seq and bulk RNA‐seq were commonly employed to capture these gene expression profiles, offering high‐resolution insights into *Plasmodium* and host responses to herbal interventions. For instance, Kane et al. [35], using *P. falciparum* cultures (W2 and D6) with a parasitaemia level of 4%, utilised RNA‐seq to assess the impact of *A. afra* extracts on *Fab_I* (ENR), *Fab_Z* (β‐hydroxyacyl‐ACP dehydratase) and *Actin* (housekeeping) genes, revealing disruptions in fatty acid biosynthesis pathways critical to parasite survival. Challenges in these studies included data variability due to differences in sample quality, RNA extraction methods and sequencing depth, as well as complexities in bioinformatics pipelines for data normalisation and differential expression analysis. Variability in herbal extract composition, as seen with methanolic extracts of *T. albida* or *Artemisia* species, further complicates reproducibility across studies. Despite these challenges, the identification of key genes and pathways, such as those involved in apoptosis [15], inflammation [17] and parasite development [18], underscores the potential of gene expression profiling to elucidate the molecular mechanisms of herbal interventions in malaria research. While the reviewed studies highlight the regulatory potential of herbal compounds on both host and parasite gene expression, an important yet underexplored opportunity lies in systematically linking these transcriptional changes to phenotypic outcomes using integrative approaches. For instance, correlating differential expression patterns with parasite clearance rates, cytokine profiles or survival outcomes could validate the functional relevance of these transcriptional shifts. Moreover, single‐cell RNA‐seq offers a promising avenue to resolve intra‐population heterogeneity in *Plasmodium* responses to natural products, which bulk RNA‐seq may mask. Going forward, coupling gene expression data with metabolomic or proteomic readouts could provide a more holistic understanding of the molecular cascades influenced by herbal interventions, potentially revealing synergistic effects or compensatory mechanisms that underlie treatment efficacy. Such integration would not only enrich mechanistic insight but also enhance the translational value of gene expression profiling in antimalarial research.

### 3.3. Molecular Docking for Drug‐Target Interactions

Computational tools, including AutoDock, AutoDock Vina, PyRx, Schrödinger and Molegro Virtual Docker, were used to predict interactions between phytochemicals and well‐defined *Plasmodium* protein targets, primarily *P. falciparum* enzymes such as *PfLDH* (1CET, 1LDG, 1T2C, 1U5A), *Falcipain-2* (3BPF, 6JW9, 7E10), *Plasmepsin II* (1LF3, 2BJU, 2IGY, 4CKU, 4YA8), *DHFR–TS* (1J3I, 2BL9, 3UM8), *PfDHODH* (1TV5, 5TBO, 3I65) and the 20S proteasome (7LXU), with species, strain and structural identifiers detailed in Tables 3 and 4. Among the most frequently targeted proteins, *P. falciparum* lactate dehydrogenase (*PfLDH*, PDB IDs: 1CET, 1LDG, 1T2C, 1U5A) was prominent, with a targeting frequency of approximately 21.43% across the dataset, as evidenced by its mention in 12 studies. In all included studies, docking was performed against structurally defined targets, most commonly *P. falciparum* proteins such as *PfLDH* (e.g., PDB IDs 1CET, 1LDG, 1T2C, 1U5A), *Falcipain-2* (3BPF, 6JW9, 7E10), *Falcipain-3* (3BWK), *Plasmepsin I* (3QRV, 3QS1), *Plasmepsin II* (1LF3, 2BJU, 2IGY, 4CKU, 4YA8), *DHFR–TS* (1J3I, 2BL9, 3UM8), *PfDHODH* (1TV5, 5TBO, 3I65) and the 20S proteasome (7LXU). *P. falciparum* 3D7 structures were used as reference models, even when the biological focus included other *Plasmodium* species such as *P. vivax* or *P. berghei*, consistent with the high sequence and functional conservation of these enzymes across species.

**TABLE 3:** Binding affinities and experimental data for the best nonstandard drug compounds.

Compound	Binding affinity (kcal/mol)	Target protein	Experimental IC_50_	References
Isorhamnetin	−156.333	Plasmepsin II (2BJU)	NA	[65]
Euphorbianin	−151.001	Plasmepsin II (2BJU)	NA	[65]
Afzelin	−143.385	Plasmepsin II (2BJU)	NA	[65]
Myricitrin	−137.29	Plasmepsin II (2BJU)	NA	[65]
Quercitrin	−132.938	Plasmepsin I (3QRV)	NA	[65]
Leucocyanidol	−115.458	Plasmepsin II (2BJU)	NA	[65]
Pinocembrin	−98.8994	Plasmepsin II (2BJU)	NA	[65]
11‐O‐Galloylbergenin	−16.22	PfLDH (Not specified)	7.85 A ± 0.61 AµM	[21]
Myricetin 3‐O‐glucoside	−13.413	PfLDH (1T2C)	NA	[36]
2″‐O‐Galloylisovitexin	−12.896	PfLDH (1T2C)	NA	[36]
Bergenin	−12.13	PfLDH (Not specified)	6.92 Â± 0.43 ÂµM	[21]
Sitoglucoside	−11.6	pfENR (1VRW)	NA	[51]
Isovitexin	−11.485	PfDHFR–TS (3UM8)	NA	[66]
Rutin	−11.25	AMA1 (3SRI), M18AAP (6PEV), Pf12p (7KJ7), TyrRS (3VGJ)	NA	[60]
Astragalin	−11.02	AMA1 (3SRI), M18AAP (6PEV), Pf12p (7KJ7), TyrRS (3VGJ)	NA	[60]
Ergosterol peroxide	−10.9	pfENR (1VRW)	NA	[46, 51]
Friedelin	−10.6	DHFR (2BL9)	NA	[46]
Vitexin	−10.601	PfDHFR–TS (3UM8)	NA	[66]
Bauerenol	−10.5	DHFR (2BL9)	NA	[46]
Cynaroside	−10.5	LysRS (4YCV)	NA	[46]
Quercetin 3‐O‐alpha‐L‐rhamnopyranoside	−10.45	AMA1 (3SRI), M18AAP (6PEV), Pf12p (7KJ7), TyrRS (3VGJ)	NA	[60]
Î^2^‐Sitosterol	−10.2	pfENR (1VRW)	734.3 Â± 1.213 Âµg/mL	[51]
Sesamin	−10	PfDHODH (5TBO)	> 10,000 nM	[50]
Isoquercitrin	−9.93	AMA1 (3SRI), M18AAP (6PEV), Pf12p (7KJ7), TyrRS (3VGJ)	NA	[60]
Sophoroside	−9.711	PfDHFR–TS (3UM8)	NA	[66]
Alpha‐muurolene	−9.7	mJHBP (5V13)	NA	[34]
Gamma‐cadinene	−9.7	mJHBP (5V13)	NA	[34]
Artocarpin	−9.6	Falcipain‐2 (3BPF)	NA	[48]
Pyrogallol	−9.5	Falcipain‐2 (3BPF)	NA	[48]
Decarine	−9.5	PfDHODH (5TBO)	1333 nM (with Trans‐fagaramide)	[50]
Luteolin	−9.5	EphA2 (6FNH)	3.4 ÂµM	[64]
Epicatechin‐gallate	−9.3	PfCDPK‐1 (I‐TASSER model)	NA	[19]
2‐Ethylacridine	−9.3	PfDHODH (3165)	NA	[31]
Rhaponticin	−9.3	PfDHODH (5TBO)	NA	[54]
Ascorbic acid	−9.3	Falcipain‐3 (3BWK)	NA	[48]
Isocolumbin	−9.3	EphA2 (6FNH)	NA	[64]
Pachyrrhizin	−9.9	Hexkinase‐1 (1CZA)	NA	[61]
Ursolic acid	−9.7	PfEMP‐1 (7JGD)	NA	[45]
Betulinic acid	−9.1	PfPKG (5DYL)	NA	[45]
Picracin	−9.1	PfCDPK‐1 (I‐TASSER model)	NA	[19]

Abbreviation: NA, not available.

**TABLE 4:** Target proteins in malaria research grouped by biological function.

Biological function	Target protein	PDB ID/status	References
Proteases	Falcipain‐2 (FP2)	3BPF, 6JW9, 7e10	[47, 48, 52, 67]
	Falcipain‐3 (FP3)	3BWK	[26]
	M18 aspartyl aminopeptidase	6PEV	[60]
	Plasmepsin I	3QS1, 3QRV	[33, 65]
	Plasmepsin II	1LF3, 2BJU, 2IGY, 4YA8, 4CKU, 5JOD	[47, 48, 59, 65, 68]
	Plasmepsin IV	5JOD	[65]
	Plasmepsin X (PMX)	7RY7	[69]
	20S proteasome	7LXU	[57]

Metabolic enzymes	Dihydrofolate reductase (DHFR)	2BL9	[32, 46]
	Dihydrofolate reductase–thymidylate synthase (DHFR–TS)	1J3I, 3UM8, 113K	[28, 53, 66]
	Dihydroorotate dehydrogenase (PfDHODH)	5TBO, 1TV5, 3165	[21, 31, 42, 50, 54]
	Enoyl acyl‐carrier protein reductase (pfENR)	1VRW	[51]
	Fructose‐bisphosphate aldolase	PF3D7_1444800 (no PDB ID)	[70]
	Hexokinase‐1	1CZA	[61]
	Lactate dehydrogenase (PfLDH)	1LDG, 1U5A, 1T2C, 1CET, 1U40, no PDB ID	[23, 27, 29, 36, 40, 43, 47, 49, 68, 71–73]
	LDH/GAPDH	PF3D7_1320800/PF3D7_1462800 (no PDB ID)	[70]
	Phosphoethanolamine methyltransferase (PMT)	3UJB, 2UJ9	[28, 47]
	Phosphocholine cytidylyltransferase	4ZCS, 4ZCR	[31, 57]
	Spermidine synthase	4CWA	[31]
	Choline kinase	6YXT	[24]
	Hypoxanthine‐guanine‐xanthine phosphoribosyltransferase (PfHGXPRT)	1TUX	[74]
	Glyoxalase‐1 (GLO‐1)	No PDB ID specified	[49]
	Phosphoglycerate kinase (PGK)	PF3D7_0922500 (no PDB ID)	[70]

Transport proteins	Ca^2+^‐ATPase	2KNE	[38]
	Calcium transporter CAX	4K1C	[38]
	Mosquito juvenile hormone‐binding protein (mJHBP)	5V13	[34]
	PfATP6	Homology modelled	[39]

Signalling proteins	Aurora A	4O0U	[25]
	Aurora B	4AF3	[25]
	EphA2 receptor	6FNH, 5NK0	[64]
	PfCDPK‐1	No PDB ID modelled via I‐TASSER	[19]
	PfPKG (cGMP‐dependent protein kinase)	5DYL	[45]
	PfCLK1, PfCLK4, PfMap2, PfNek1	Homology models	[56]

tRNA synthetases	Anopheles gambiae bifunctional glutamyl/prolyl‐tRNA synthetase (AgEPRS)	No PDB ID homology modelled	[37]
	Glutamyl‐tRNA synthetase (ERS)	7WAJ	[69]
	Lysyl‐tRNA synthetase	4YCV	[46]
	Tyrosyl‐tRNA synthetase	3VGJ	[60]
	PfcPRS	PF3D7_1213800 modelled on human PRS (4HVC)	[75]

Chaperones/stress response	Anopheles gambiae heat shock protein 70 kDa (AgHSP70KDa)	No PDB ID homology modelled	[37]
	Hsp70‐1 (PfHsp70‐1)	Homology model no PDB ID; PF3D7_0818900	[22, 76]
	Hsp70‐z (PfHsp70‐z)	No PDB ID	[33]

Antioxidant enzymes	Superoxide dismutase (SOD1)	2C9V	[26]
	Catalase (CAT)	1QQW	[26]
	Glutathione peroxidase (GPx1)	1GP1	[26]
	Glutathione reductase (GR)	1XAN	[26]

DNA/RNA processing	Apicoplast DNA polymerase (apPOL)	7SXQ	[69]
	DNA topoisomerase II	6CA8	[77]

Membrane/structural proteins	Apical membrane antigen 1	3SRI	[60]
	Erythrocyte membrane protein 1 (PfEMP‐1)	7JGD, 3C64	[30, 45]
	*Plasmodium falciparum* 6‐cysteine s48/45 domain	2LOE	[78]
	Pf12p	7KJ7	[60]
	Pfg27 (gametocyte protein)	No PDB ID	[23]
	PCM1	PF3D7_1120100 (no PDB ID)	[70]

Electron transport	Cytochrome bcl reductase	4PD4	[57]
	Cytochrome c2 (cyt c2)	7TXE	[67, 69]
	Cytochrome c2 domain‐swapped dimer (cyt c2 DSD)	7TXE	[21]

Immune/inflammatory response	High mobility group Box 1 (PfHMGB1 HsHMGB1)	2MRC, 1HME	[49]
	IL6	No PDB ID	[14]
	TNF Alpha	No PDB ID	[79]

Haemozoin formation	Haemozoin crystal lattice	No PDB ID specified	[77]

Ion channel	hERG	5VA2	[55]

Other key targets included *Dihydrofolate Reductase–Thymidylate Synthase* (DHFR–TS, 1J3I, 2BL9, 3UM8), *Dihydroorotate Dehydrogenase* (*PfDHODH*, 1TV5, 5TBO, 3165) and 20S proteasome (7LXU). For example, Samuel et al. [36] reported myricetin 3‐O‐glucoside with a binding affinity of −13.413 kcal/mol against *PfLDH* (1T2C), validated through in silico pharmacophore modelling and ADMET predictions. Similarly, Elmaidomy et al. [57] identified limonin, luteolin and myricetin with ΔG_binding < −7 kcal/mol for *20S proteasome* (7LXU), c*holine kinase* (6YXT) and *phosphocholine cytidylyltransferase* (4ZCS), confirmed by in vitro Malstat assays and MDs simulations using Desmond. Chaurasia and Pandey [48] demonstrated strong binding of artocarpin (−9.6 kcal/mol) to *Falcipain-2* (3BPF) and ascorbic acid (−9.3 kcal/mol) to *Falcipain-3* (3BWK) among 50 phytochemicals from *Artocarpus* species, supported by in vitro β‐haematin assays (91.7% efficiency compared to artemisinin). Singh et al. [60] reported rutin’s exceptional affinity (−11.25 kcal/mol) for *Apical Membrane Antigen 1* (3SRI), validated through in vitro parasitaemia reduction and hemocompatibility assays. Dzouemo et al. [50] found sesamin (−10.0 kcal/mol) and decarine (−9.5 kcal/mol) binding strongly to *PfDHODH* (5TBO), corroborated by in vitro antiplasmodial assays and spectroscopic data. Binding affinities across studies typically ranged from −6.0 to −13.4 kcal/mol, with notable outliers like euphorbianin (−151.001 kcal/mol) against *Plasmepsin II* (2BJU) in Shah et al. [65], validated via Lipinski’s rule and ADMET profiling. Where *P. falciparum* structures were used as surrogates for orthologous proteins from other *Plasmodium* species, this choice was supported by reported high sequence identity and conservation of active‐site residues (e.g., *PfLDH*, *DHFR–TS*, *PfDHODH*), indicating that the docking poses are likely to be biologically relevant across species.

Validation of these docking predictions was robustly conducted using a variety of in vitro and in vivo methods to ensure reliability. Several studies combined docking with in vitro, in vivo and cytotoxicity profiling of nonstandard compounds, providing more translational evidence for their antimalarial potential (e.g., β‐carboline derivatives and plant‐derived scaffolds) [80–82]. In vitro assays, including Malstat, SYBR Green I, *pLDH*, β‐haematin inhibition and luciferase assays, were frequently utilised, most commonly against *P. falciparum* laboratory strains such as 3D7 (chloroquine‐sensitive), NF54 and W2 or K1 (chloroquine‐resistant), as well as rodent models like *P. berghei* ANKA where applicable, as reported by Hidayati et al. [52], Adeoye et al. [38] and Mianda et al. [77]. However, not all studies consistently reported the *Plasmodium* strain used, which may complicate comparisons given known strain‐dependent differences in drug response. In vivo validations, such as parasitaemia suppression, survival curves and Peters’ tests, were reported by Asanga et al. [45], Herman et al. [75] and Alebachew et al. [68]. Additional methods included spectroscopic analyses (NMR, MS, FTIR), cytotoxicity assays (MTT, LDH) and MD simulations assessing RMSD, RMSF and hydrogen bond profiles, as noted in Rawa et al. [27] and Chaurasia and Pandey [48]. Computational validations, such as redocking with RMSD < 2.0 Å, were reported by Adams et al. [53] (RMSD = 0.959 Å). Ramachandran plots and ADME–Tox profiling were also reported by Shekari et al. [37] to further strengthen the findings. For instance, Konyanee et al. [43] validated rheediachromenoxanthone’s binding (−8.6 kcal/mol) to *PfLDH* (1LDG) through in vitro IC_50_ determination and redocking (RnMSD = 0.775 Å).

Although these developments were made, there has been a constraint on the accuracy of the calculations and experimental validation. Such dependence on homology models as *PfCDPK-1* [19] or *Anopheles gambiae HSP70KDa* [37] created uncertainty because of possible errors in the binding sites predicted. Due to the variation between AutoDock Vina and Glide docking algorithms, it was observed that the binding affinity estimates were not consistent, as reported by Shah et al. [65]. The small sample sizes and the variations in the composition of the herbal extracts [59, 71], as well as the lack of clinical translation, were also among the challenges in the field of experiments, as Onguéné et al. [20] emphasised. Drug development is further complicated by the complexity of targeting proteins that are specific to a particular stage, for example, *Pfg27* in gametocytes [23]. These limitations indicate the importance of standardised docking methods, improved homology modelling methods and thorough in vivo experiments in applying computational predictions to effective antimalarial therapies.

Molecular docking has succeeded in establishing the strong binding effects of phytochemicals with the *Plasmodium* targets, but much more is still unknown about the mechanism that causes effective inhibition in the biological context of the parasite. Numerous experiments aim to only predict the strength of binding, without follow‐up experiments or kinetic modelling to determine the duration of binding or the selectivity of action. Further, the majority of docking studies use constant protein structures, ignoring the situation in living organisms where binding can be affected due to their dynamic and flexible conditions. The accuracy can be enhanced with the use of sophisticated approaches such as ensemble docking, induced‐fit simulations and ML‐based refinement of binding predictions. Moreover, the compounds that interfere with several important parasite processes, including glycolysis and proteasome, should become the priority to increase the treatment efficacy and decrease the chances of resistance. Implementing these improvements would transform molecular docking into a more reliable tool for developing new antimalarial therapies.

#### 3.3.1. Docking Studies of Nonstandard Drug Compounds Against *Plasmodium* spp. Targets

**FIGURE 2:**
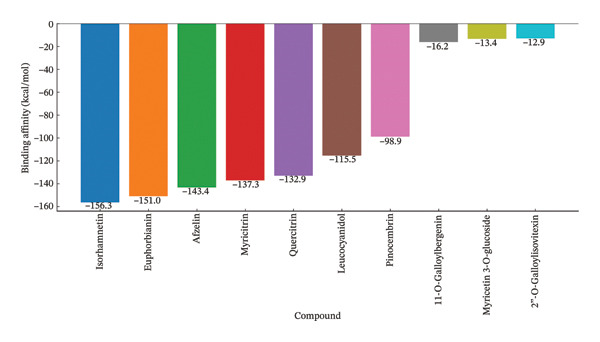
Binding affinities of top 10 nonstandard drug compounds to antimalarial targets.

Molecular docking studies have identified several nonstandard drug compounds with promising binding affinities (ΔG, kcal/mol) against *P. falciparum* and related targets, offering potential leads for antimalarial drug development. Table 3 summarises the binding affinities, target proteins, experimental IC_50_ values and sample sizes for 40 nonstandard compounds reported in studies from 2014 to 2024, excluding standard drugs such as chloroquine and artemisinin. Binding affinities for the top‐ranked nonstandard compounds ranged from −156.333 kcal/mol for isorhamnetin to −86.0838 kcal/mol for scoparone, with euphorbianin (−151.001 kcal/mol), afzelin (−143.385 kcal/mol), myricitrin (−137.29 kcal/mol), quercitrin (−132.938 kcal/mol), leucocyanidol (−115.458 kcal/mol), artemisinin (−101.408 kcal/mol), pinocembrin (−98.8994 kcal/mol) and scopoletin (−98.4147 kcal/mol) occupying intermediate positions in this high‐affinity range [65]. Figure 2 illustrates the binding affinities of the top 10 compounds, highlighting the dominance of *Plasmepsin II* and *PfLDH* as targets. Notably, several of these ligands, particularly isorhamnetin, euphorbianin and afzelin, target *Plasmepsin II* and exhibit markedly more negative binding energies than most other screened compounds, indicating exceptionally strong predicted interactions but also highlighting the need for cautious interpretation because such extreme values often arise from MM–PBSA‐type rescoring and are sensitive to methodological differences across studies. Other notable compounds include 11‐O‐galloylbergenin (−16.22 kcal/mol, *PfLDH*) [21], myricetin 3‐O‐glucoside (−13.413 kcal/mol, *PfLDH*) [36] and isovitexin (−11.485 kcal/mol, *PfDHFR–TS*) [66]. Flavonoids and glycosides, such as rutin (−11.25 kcal/mol), astragalin (−11.02 kcal/mol) and isoquercitrin (−9.93 kcal/mol), were frequently among the stronger binders, indicating their promise as antimalarial candidates [60].

Target proteins were quite variable, with the most common being *PfLDH*, *Plasmepsin II* and *PfDHFR–TS*; see Table 4. Compounds based on *Plasmepsin II* showed maximum affinities [65] and *PfLDH*‐targeting compounds such as 11‐O‐galloylbergenin and bergenin have also shown promising binding properties and experimental IC_50_ values (7.85 ± 0.61 and 6.92 ± 0.43 µM, respectively), indicating the antiviral potentials [21]. Few works reported IC_50_ values, which makes it difficult to correlate docking scores and experimental efficacy. Sample sizes were reported sparsely, with some notable exceptions for isovitexin (*n* = 81), rutin (*n* = 3) [60] and friedelin (*n* = 5) [46], just to name a few. Figure 2 shows the binding affinities of the top 10 compounds with the dominance of *Plasmepsin II* and *PfLDH* as targets. The variability in docking methodologies (AutoDock, MM–PBSA) and nonstandard deviation and nonstandard error data are challenges for direct comparisons. Nevertheless, these findings highlight the potential of flavonoids, glycosides and other nonstandard compounds for further study in antimalarial drug development.

### 3.4. ML in Drug Resistance and Discovery

**FIGURE 3:**
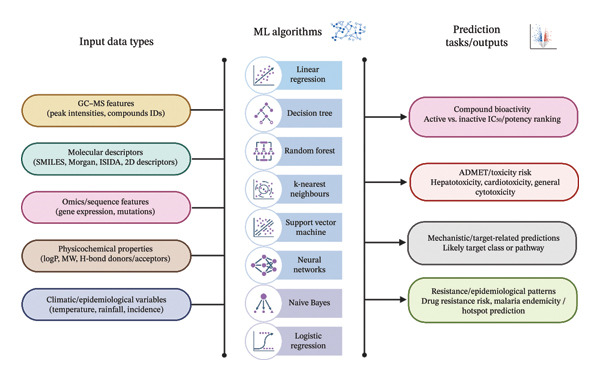
Visual overview of machine learning algorithms in antimalarial research.

ML models, such as RF, SVM, k‐nearest neighbours (KNN), logistic regression (LR), decision trees (DT), Chemprop and genetic function algorithms (GFA), have made significant contributions to the study of malaria by analysing genomic information and determining the resistance of pathogens to drugs, in this case *Plasmodium.* To show how these approaches are implemented in studies, Figure 3 is a visual summary of the most commonly used ML algorithms found in this review. Table 5 summarises the ML models for bioactivity prediction with input data, output and performance. These models have also helped in the discovery of new antimalarial compounds from herbal sources. For example, Deligianni et al. [15] reported that KNN, LR and DT models were used to predict the bioactivity of the compounds in 239 components of *Euphorbia hirta* essential oils (EOs); the best results were 0.701–0.840 MCC based on leave‐some‐out cross‐validation classification. Top predictive compounds, including eugenol, eucalyptol and limonene, were identified based on consensus weighted feature importance (WFI) analysis, with the robustness of our models validated by ROC AUC/PR curves (best MCC: 0.840). Similarly, Miao et al. [14] used Chemprop and MAIP models along with SMILES and Morgan fingerprints from *Morus alba* S1 methanolic leaf extract that yielded 0.868590 +‐ 0.007887 for the extent of probability of antimalarial. Potential based on flavonoids such as quercetin and kaempferol. Oyebamiji et al. [72] used GFA to model QSAR of *C. myxa* phytochemicals using 2D descriptors (e.g., ALogP2, ATS1m) for the prediction of binding affinities with a good R2 of 0.806 and cross‐validation R2 of 0.612. Viira et al. [41] developed SVM classification consensus models for curcuminoid derivatives using SMILES and ISIDA fragment descriptor resolutions to obtain cross‐validated balanced accuracy at 0.701–0.953, with 100% sensitivity in prospective predictions of 17 active compounds. Pradhan et al. [32] used the binary LR algorithm to predict the endemicity of malaria using climatic variables, and the results demonstrated the versatility of ML for an epidemiological application.

**TABLE 5:** Summary of machine learning models for bioactivity prediction with input data, outputs and performance metrics.

Machine learning model	Input data type	Prediction output	AUC/accuracy	References
KNN, logistic regression, decision tree	GC–MS data (239 components), chemical composition	% inhibition of zygote/ookinete formation	MCC: 0.701–0.840, Best MCC: 0.840	[15]
Binary logistic regression	Climatic variables (temperature, humidity, rainfall, elevation)	Endemicity prediction (1 = endemic, 0 = non‐endemic)	Not specified	[32]
Chemprop, MAIP	Molecular structures (SMILES, Morgan fingerprints)	Antimalarial activity probability	AUC: 0.868590 ± 0.007887	[14]
Genetic function algorithm (GFA)	2D descriptors (ALogP2, ATS1m, ATS7m, ATS0v)	Binding affinity (QSAR model)	*R* ^2^ = 0.806, cross‐validated *R* ^2^ = 0.612	[72]
Random forest (RF)	Chemical descriptors (logP, drug likeness, amines, etc.)	Cardiotoxicity, hepatotoxicity, mutagenicity	AUC: 0.830–0.869	[55]
Support vector machine (SVM)	Molecular structures (SMILES), ISIDA fragment descriptors (39 sets)	Binary classification (active/inactive)	Balanced accuracy: 0.701–0.953, prospective: 0.62	[41]

In drug discovery, ML models have played a predominant role in discovering novel compounds of herbal interventions. For instance, Mianda et al. [77] combined ML with UPLC–QTOF–MS data from *Aloe marlothii* root extracts, and the molecular targets, such as *DNA topoisomerase II*, were predicted (PDB: 6CA8), and aloesaponarin I and aloesaponol IV, which showed the best IC_50_ values and binding affinities, indicated they are strong inhibitors. Rehman [42] applied network pharmacology and ML to the *Xanthium cavanillesii* ethanolic extracts, by prediction of binding energy and enriched pathways (e.g., pyrimidine metabolism) for artemisinin derivatives against *PfDHODH* (1TV5), also confirmed by ITC measurements. Olaosebikan et al. [31] and Brahma et al. [67] used ML to assess the *Andrographis paniculata* and *Hibiscus cannabinus*/*Corchorus capsularis* extracts, respectively, based on GC–MS data, such as predicting binding affinities and ADMET properties and identifying compounds, for example, andrographolide and sesamin. Kadioglu et al. [55] used RF to predict the toxicity profile of artemisinin derivatives from *Artemisia* species, and the achieved AUC values were 0.830–0.869 for cardiotoxicity and hepatotoxicity models. These studies highlight the usefulness of ML in the prioritisation of phytochemicals with high potential for antimalarial application, which are typically confirmed through in vitro assays (i.e., luciferase and SYBR Green I), as well as the use of in silico docking.

Despite these advancements, there are still challenges in the applications of ML. Data availability has also been a major challenge, as data from several studies have been based on a limited or heterogeneous type of data (GC–MS profiles, SMILES strings, etc.), probably insufficient to reflect the complexity of *Plasmodium* biology [29]. Model interpretability is another issue, especially in the case of complex models such as SVM and RF, where feature importance (WFI) [15] has to be carefully validated, and drivers/importances of features have to be strictly validated to avoid misinterpretation. Overfitting is a recurrent problem, particularly for QSAR models with high‐dimensional descriptors, such as in the case of Oyebamiji et al. [72], where cross validation was very important to make the model generalisable. Additionally, the combination of genomic data for resistance prediction, such as that attempted by Herman et al. [75] for *PfcPRS* mutations, is constrained by the lack of well collateralisation of resistance data sets. These challenges highlight an urgent need for larger, standardised datasets, better feature selection techniques and hybrid strategies that combine ML with molecular docking and dynamics to better predict and interpret malaria drug discovery.

ML has enhanced the selection of promising compounds and prediction of resistance in research on malaria, but this approach is limited due to a lack of data availability, unclear outcomes and a lack of biological context. Many ML models rely on simplified forms of chemical data, such as SMILES or even 2D fingerprints or computationally derived fingerprints, which do not truly capture biological factors like protein shapes or host–parasite interactions. To mitigate this, future approaches should integrate chemical, genetic and biological information into unified ML systems. For example, advanced models such as graph neural networks and transformer models are better able to model more complicated interactions in molecules; at the same time, explainable AI can help us understand what drives predictions to better validate experimental results. In addition, up‐to‐date training models using a variety of *Plasmodium* strains and data from other parasite life stages might render predictions more reliable, especially for the emergence of resistance. In the future, the combination of ML and active learning systems that could be adapted according to the new biological data could make ML a dynamic tool for finding new antimalarial treatments.

### 3.5. Pathways Modulated by Molecular Techniques in Malaria Research

Molecular techniques, including gene expression profiling, molecular docking and ML, have significantly advanced the understanding of *Plasmodium* pathways targeted by phytochemicals and synthetic compounds, offering insights into antimalarial drug development [83, 84]. Table 6 summarises the affected pathways and their respective validation methods. One of the most frequently targeted pathways is *PfLDH* inhibition, critical for parasite survival within erythrocytes. Shah et al. [65] demonstrated that euphorbianin (−151.001 kcal/mol) and isorhamnetin (−156.333 kcal/mol) potently inhibit *Plasmepsin II* (2BJU), disrupting haemoglobin digestion in the parasite’s food vacuole, validated through ADMET profiling and Ramachandran plots. Chaurasia and Pandey [48] reported artocarpin’s strong binding (−9.6 kcal/mol) to *Falcipain-2* (3BPF), confirmed by in vitro β‐haematin assays, which also inhibit hemozoin formation, a key detoxification process. Aini et al. [69] identified quercetin’s inhibition of *Plasmepsin X* (7RY7, −7.8 kcal/mol), further emphasising haemoglobin degradation as a therapeutic target. Mianda et al. [77] showed that *aloesaponarin I* from *Aloe marlothii* inhibits β‐haematin polymerisation in the asexual blood stage, validated by luciferase assays, while Wiraswati et al. [33] highlighted *Plasmepsin I* inhibition by *Breynia cernua* extracts.

**TABLE 6:** A summary table of the affected pathways and their respective validation methods.

S/No.	Affected pathway	Validation methods	References
1	Immune response (inflammation, T cell recruitment, phagocytosis)	RT‐qPCR (gene expression: TNF, IL‐1β, IL‐6, IL‐12, IFNγ), histological analysis, TUNEL assay (apoptosis), in vivo parasitaemia suppression, LDH assay (cytotoxicity), EC_50_ (antioxidant), HRMS/MS (phytochemicals)	[44, 62]
2	Oxidative stress (GSH/CAT/MDA/NO, ROS, HO‐1)	Biochemical assays (GSH, CAT, MDA, NO), RT‐qPCR (Bcl2/Bax/caspase‐3), histopathology, TEAC (ABTS assay), GSH (DTNB assay), TBARS (lipid peroxidation), in vivo parasitaemia suppression, microscopy (Giemsa‐stained smears)	[26, 58, 62]
3	Lysyl‐tRNA synthetase (LysRS)	Molecular docking (AutoDock Vina), ADMET prediction (SwissADME), in vivo parasitaemia suppression, haematological indices	[46]
4	Dihydrofolate reductase (DHFR) pathway	Molecular docking (AutoDock Vina), ADMET prediction (SwissADME), in vivo parasitaemia suppression, dose‐response curves (nonlinear regression), microscopic parasite clearance, ROC curve (AUC = 0.733), RMSD (0.959 Å)	[32, 46, 53, 66]
5	Plasmepsin I inhibition (haemoglobin degradation)	In vitro antiplasmodial assay (HRP2 ELISA), molecular docking (AutoDock Vina), Ramachandran plot, ProSA‐web z‐score, RMSD comparison (I‐TASSER, Rosettafold)	[19, 33, 59, 65]
6	Amino acid starvation response (AAR, eIF2*α* phosphorylation, PfcPRS inhibition)	Whole‐genome sequencing (SNPs via Illumina, SAMtools), yeast complementation, Western blot (eIF2*α* phosphorylation), enzymatic assays (K_i_, K_m_), Peters’ test (> 99% parasitaemia reduction), molecular modelling (AMBER, Glide)	[75]
7	Anaerobic glycolysis (PfLDH inhibition, ATP synthesis)	Molecular docking (AutoDock Vina, RMSD ∼2.0 Å), MD simulation (RMSD, RMSF, Rg, H‐bonds), MM–PBSA, pLDH assay, IC_50_ determination, NMR/HR‐MS, redocking (NADH, RMSD = 0.775 Å), Moldock score, pKi	[23, 27, 40, 43, 70–72]
8	Antioxidant defence (SOD, CAT, GPx, GR)	Repeated oxidative stress measurements (TEAC, GSH, TBARS), molecular docking (Molegro Virtual Docker), statistical significance (*p* ≤ 0.05), NMR compound identification	[26]
9	Apicoplast DNA replication (apPOL), Protein translation (glutamyl‐tRNA synthetase)	Molecular docking (PyRx), RMSF, Rg, Protein Plus analysis, Lipinski’s Rule of Five	[69]
10	Haemozoin formation (β‐haematin inhibition)	In vitro β‐haematin assay, luciferase assay (gametocyte viability), SYBR Green I assay (ABS parasite growth), molecular docking (Schrödinger, AutoDock 4.2), UPLC–QTOF–MS, HMBC, COSY, comparison with artemisinin	[38, 48, 51, 52, 77, 85]
11	Cardiotoxicity (hERG, gene expression)	In vitro (AC16 cardiomyocytes), in vivo (zebrafish), molecular docking, microarray	[55]
12	Cholesterol sequestration, lipid raft disorganisation, HMGB1, GLO‐1	In vitro pLDH assay (IC_50_), in vivo parasitaemia clearance, NMR (PfHMGB1, GLR‐HsHMGB1), molecular docking (ΔE, ΔG), MTT assay (Vero cells, SI = 9.6)	[49]
13	Dihydroorotate dehydrogenase (DHODH)	In vitro schizont maturation inhibition, molecular docking (AutoDock Vina, RMSD = 0.15 Å), ADMETlab 2.0, IC_50_ values, selectivity index (SI)	[31, 54]
14	Cysteine/methionine‐rich protein functions (M18, Tyrosyl‐tRNA synthetase, Pf12p)	Molecular docking, in vitro parasitaemia reduction (Giemsa smears), IC_50_, hemocompatibility assay, MTT assay (HEK293), physicochemical analysis (UV–vis, DLS, Zeta, FTIR, XRD, FESEM)	[15, 60, 77]
15	Gametocyte‐to‐ookinete development	In vitro inhibition (> 50%, *p* ≤ 0.01), luciferase assay, fluorescence microscopy (Pfs25 antibody), molecular docking (Schrödinger), SEA/SuperPred target prediction, statistical thresholds (CR inhibition > 50%)	[15, 77]
16	Erythrocyte invasion (PfCDPK‐1)	Molecular docking, Ramachandran plot, ProSA‐web z‐score, RMSD comparison (I‐TASSER, Rosettafold), in vitro assays	[19, 35]
17	Fatty acid biosynthesis (FAS‐II)	qPCR (gene expression), GC–MS, molecular docking (binding affinity)	[35]
18	Folate metabolism (DHFR–TS)	Molecular docking (Glide, PyRx), ROC curve (AUC = 0.733), RMSD (0.959 Å), correlation with pIC_50_ (*r* ^2^ = 0.8374), redocking cycloguanil	[53, 66]
19	Glycolysis (hexokinase‐1, PCM1, GAPDH, Aldolase, PGK), purine recycling	In silico validation (PROCHECK, ERRAT, PASS), molecular docking, QSAR (*R* ^2^ = 0.806), MD simulation (100 ns), 2‐DE, LC/MS/MS, PIQUES™ software	[61, 70, 72]
20	Proteasome degradation	Protein spot quantification (≥ 2‐fold up‐regulation), PIQUES™ software, 2‐DE, LC/MS/MS	[70]
21	Mitochondrial electron transport (cytochrome c2, bc1)	Molecular docking (AutoDock Vina), PASS (Pa: 0.892), MD simulation, NMR, HRESIMS, Malstat assay, in vitro parasite growth inhibition, haemolytic activity, cytotoxicity (A549, Vero)	[24, 67, 69]
22	PfHsp70‐1 chaperone function, protein folding	In vitro UV–Vis spectroscopy, MDH aggregation suppression assay, pLDH assay, ATPase assay (normalised to basal activity), SDS–PAGE, comparison with chloroquine (IC_50_ = 8.5 ng/mL)	[22, 76]
23	Purine salvage pathway (PfHGXPRT)	In vitro pLDH assay, cytotoxicity (MTT), biophysical assays (DSF, UV–vis), molecular docking	[74]
24	Pyrimidine metabolism (UMP biosynthesis, DHODH)	Molecular docking (AutoDock Vina, RMSD = 0.15 Å), ITC (K_a_, ΔH), network pharmacology (STRING DB, *p* = 6.86e‐09), pathway enrichment (FDR < 0.001), ADMETlab 2.0, in vitro IC_50_	[31, 42]
25	NF‐κB pathway, endothelial apoptosis	Molecular docking, binding interactions (hydrogen bonds, hydrophobic), computational physicochemical analysis	[64]
26	PfATP6 (Ca^2+^‐ATPase) inhibition	Comparative homology modelling, molecular docking, in vitro β‐haematin assay	[39]
27	PfEMP‐1 mediated adhesion, PfPKG Signalling	In vivo parasite density, % growth inhibition, GC–MS, molecular docking, ADME analysis	[45]
28	Insect hormonal regulation (mJHBP)	Molecular docking, MD simulation (RMSD, RMSF, Rg, SASA)	[34]
29	Thioredoxin reductase (TrxR), glutathione metabolism, ROS induction	SVM model (cross‐validation, balanced accuracy > 0.7), IC_50_ (ICEstimator), cytotoxicity (MRC‐5, SI), in silico consensus voting (> 70% likelihood)	[41]
30	Hypolipidemic effect, metabolic enzymes (PfLDH, Plasmepsin II)	In vivo parasitaemia reduction, biochemical assays, molecular docking, MD simulation stability (RMSD, RMSF)	[47]
31	Lysosomal/endocytic signalling (Thailandine)	NMR, MS, in vitro cytotoxicity (*n* ≥ 2), antiplasmodial (*n* = 5), 1‐way ANOVA, nonlinear regression (IC_50_)	[25]
32	Ubiquitin–proteasome, glycerophospholipid metabolism, DNA replication	In vitro Malstat assay, molecular docking (AutoDock Vina), MD simulation (Desmond), PPI network (cytoscape), docking scores, ΔG_binding < −7 kcal/mol	[57]
33	Mitotic division (PfNek1, PfMap2, PfCLK1, PfCLK4), transmembrane transport	In vitro/ex vivo susceptibility assays, molecular docking (AutoDock Vina), IC_50_, selectivity index	[56]
34	PI3K‐Akt, relaxin signalling	Molecular docking, MD simulation, machine learning (Chemprop, MAIP), binding scores	[14]

Pyrimidine and folate metabolism, essential for *Plasmodium* nucleic acid synthesis, are prominent targets. Rehman [42] used network pharmacology to demonstrate artemisinin derivatives’ inhibition of *PfDHODH* (1TV5), disrupting *de novo* pyrimidine synthesis, with enriched KEGG pathways (e.g., map00240) validated by ITC measurements. Dzouemo [50] and Sikam et al. [54] reported sesamin (−10.0 kcal/mol) and rhaponticin (−9.3 kcal/mol) targeting *PfDHODH* (5TBO), supported by in vitro antiplasmodial assays. The folate pathway, mediated by *Dihydrofolate Reductase–Thymidylate Synthase* (DHFR–TS), was inhibited by isovitexin (−11.485 kcal/mol, [66]), friedelin (−10.6 kcal/mol, [46]) and dimethylmatairesinol (−8.4 kcal/mol, [53]), with validations including in vivo parasitaemia suppression and high *r* ^2^ correlations (0.8374). Al‐Huqail [28] confirmed rutin’s inhibition of *PfDHFR–TS* (1J3I), impacting phosphatidylcholine synthesis via *PfPMT*.

Glycolysis, crucial for parasite energy production, was disrupted through *PfLDH* inhibition. Samuel et al. [36] reported myricetin 3‐O‐glucoside’s binding (−13.413 kcal/mol) to *PfLDH* (1T2C), validated by pharmacophore modelling, while Konyanee et al. [43] and Chaniad et al. [40, 73] identified rheediachromenoxanthone (−8.6 kcal/mol) and 2,4,3′,5′‐tetrahydroxybibenzyl (−8.91 kcal/mol), supported by in vitro IC_50_ data. Chaijaroenkul et al. [70] detected upregulated glycolytic enzymes (e.g., PCM1, LDH, GAPDH) in response to *Garcinia mangostana* extracts, indicating compensatory mechanisms. Mitochondrial electron transport was targeted by quercetin’s inhibition of *Cytochrome c2* (7TXE, −8.2 kcal/mol) [69] and compound 2’s binding to *Cytochrome bc1* (4PD4, −8.33 kcal/mol) [24] validated by Malstat assays. Murugesan and Kaleeswaran [61] also noted pachyrrhizin’s inhibition of hexokinase‐1 (−9.9 kcal/mol), further disrupting glycolysis.

Protein translation and amino acid metabolism were disrupted by targeting synthetases. Herman et al. [75] showed halofuginone’s inhibition of *PfcPRS*, triggering the amino acid starvation response (AAR) via eIF2*α* phosphorylation, validated by yeast complementation (K_i_ = 71.1 nM). Shekari et al. [37] reported sesamin’s inhibition of *AgEPRS* (−7.4 kcal/mol), disrupting *Anopheles gambiae* protein synthesis, validated by ADME–Tox profiling. Aini et al. [69] and Apeh et al. [46] identified quercetin (−7.5 kcal/mol) and cynaroside (−10.5 kcal/mol) targeting *Glutamyl-tRNA Synthetase* (7WAJ) and *LysRS* (4YCV), respectively, impacting apicoplast translation. Chaperone‐mediated protein folding via *PfHsp70-1* and *PfHsp70-z* was inhibited by *Ziziphus mucronata* phenolics ([22, 76]), validated by pLDH assays. The purine salvage pathway was targeted by lupeol (−7.6 kcal/mol) against *PfHGXPRT* (1TUX) [74].

Immune, inflammatory and oxidative stress pathways were also modulated. Soeiro [49] showed glycyrrhizin’s disruption of *PfHMGB1*‐mediated inflammation, validated by NMR. Camara et al. [44] and Dkhil et al. [62] reported *T. albida* and *I. oblongifolia* extracts inducing *NFκB* signalling, ROS production and cytokine modulation (*TNF, IL-6*), enhancing T‐cell recruitment, confirmed by in vivo survival studies. Gomes [26] identified eleutherol’s targeting of antioxidant defences (SOD, CAT, GPx, −69.97 kcal/mol for GPx1), validated by oxidative stress assays. Fatty acid biosynthesis (FAS‐II) was disrupted by *A. afra* extracts targeting *Fab I* and *Fab Z* (Kane et al., 2023), while calcium transport via *PfATP6* was inhibited by caffeoylquinic acids [39]. Despite robust validations (e.g., RMSD < 2.0 Å, SYBR Green I assay), challenges such as homology model inaccuracies (e.g., *PfCDPK-1*) [19] and data heterogeneity underscore the need for standardised protocols to translate these findings into effective therapies.

**FIGURE 4:**
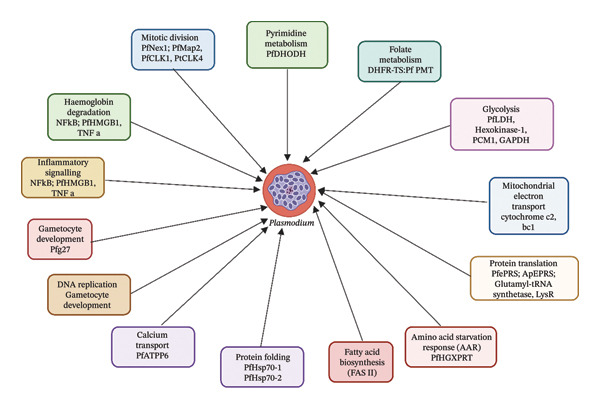
Molecular pathways targeted in *Plasmodium* by antimalarial strategies.

**FIGURE 5:**
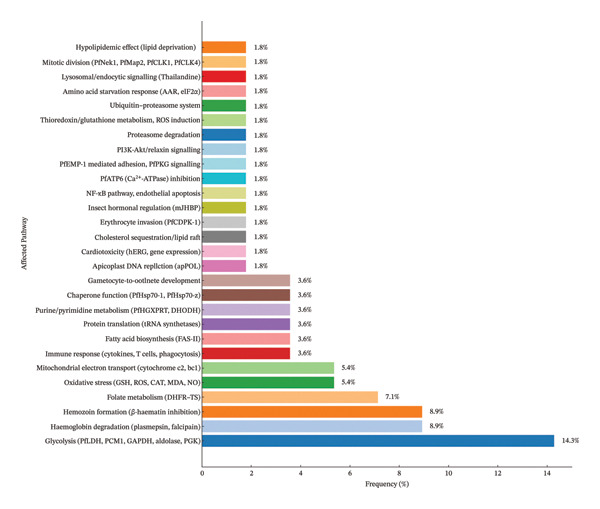
Percentage of pathways affected in malaria studies.

The various research works collectively point to a wide variety of *Plasmodium* pathways that are targeted by molecular techniques (see Figure 4), such as haemoglobin degradation (*Plasmepsin I, II, IV, X, Falcipain-2, Falcipain-3*), hemozoin formation, pyrimidine metabolism (*PfDHODH*), folate metabolism (*DHFR–TS*, *PfPMT*), glycolysis (*PfLDH*, *Hexokinase-1, PCM1, GAPDH*), mitochondrial electron transport (Cytochrome c2, bc1), protein translation (*PfcPRS*, *AgEPRS*, *Glutamyl-tRNA Synthetase, LysRS*), amino *AAR*, purine salvage (*PfHGXPRT*), fatty acid biosynthesis (*FAS-II*), calcium transport (*PfATP6*), protein folding (*PfHsp70-1*, *PfHsp70-z*), ubiquitin–proteasome system, DNA replication (*DNA topoisomerase II*, *apPOL*), gametocyte development (*Pfg27*), erythrocyte invasion (*PfCDPK-1*), mitotic division (*PfNek1*, *PfMap2*, *PfCLK1*, *PfCLK4*), inflammatory signalling (*NFκB*, *PfHMGB1*, *TNF-α*), oxidative stress (SOD, CAT, GPx, GSH) and insect hormonal regulation (*mJHBP*). These pathways range from metabolic, structural and immunological processes that are important for parasite survival and transmission, illustrating the scope of molecular control. Figure 5 shows what percentage of the pathways are affected in the studies included. The diversity of *Plasmodium* pathways that are influenced by molecular treatments highlights the importance of moving away from single‐target approaches towards a systems pharmacology approach. Studies performed by docking and gene expression have revealed key pathways such as glycolysis, pyrimidine biosynthesis and haemoglobin degradation; however, these pathways do not act independently in the complex life cycle of the parasite. Future research will have to investigate how these pathways interact and adapt, particularly in the presence of drug or immune pressure. For instance, stress on glycolysis could increase antioxidant defences or alternative energy sources with consequences for the success of a drug. Combining the data set from RNA‐seq, protein structures and metabolites with ML network models may allow prediction of resistance patterns or identify combined weaknesses of the parasite. Moving from a single‐target approach to drug design to a dynamic, networked approach would help improve the selection of effective compounds and be more appropriate for herbal treatments, which affect many pathways in both parasite and host. Ultimately, interactions among pathways instead of just identification of affected pathways are essential to developing effective, drug‐resistant antimalarial treatments.

#### 3.5.1. Strengths and Limitations

The strength of these findings lies in their good coverage of the pathway with strong validations such as in vitro assays (Malstat, SYBR Green I, β‐haematin), in vivo assays (Peters’ tests, parasitaemia suppression) and in silico metrics (RMSD < 2.0 Angstroms, *r* ^2^ = 0.8374). Molecular docking and ML have provided us with a high‐throughput screening procedure and precise identification of targets, whereas gene expression profiling has given us insight into compensatory mechanisms (upregulation of glycolytic enzymes). However, shortcomings include the use of homology models (*PfCDPK-1*) [19], which might lead to binding site inaccuracies. Data heterogeneity (variable herbal extract compositions [59, 71]) and discrepancies in docking algorithms (AutoDock Vina vs. Glide [65]) are some of the challenges to reproducibility. Limited clinical translation, according to Onguéne et al. [20], and small sample sizes in experimental validations also make therapeutic development more difficult. Addressing these limitations requires the use of standardised docking protocols, better homology modelling and larger and well‐curated data sets, which will increase the reliability and translatability of molecular results in malaria research.

## 4. Discussion

### 4.1. Integrating Multiomics and Network Pharmacology for Antimalarial Target Prioritisation

The integration of multiomics approaches, including proteomics, metabolomics and transcriptomics, with network pharmacology has emerged as a powerful strategy to prioritise antimalarial targets, addressing the complexity of *Plasmodium* biology and drug resistance [86]. Proteomics has been instrumental in validating molecular docking predictions by identifying upregulated proteins in response to phytochemical interventions. For instance, Chaijaroenkul et al. [70] used proteomics to detect elevated levels of glycolytic enzymes (PCM1, LDH, GAPDH) in *P. falciparum* treated with *G. mangostana* extracts, suggesting compensatory metabolic adaptations that align with docking studies targeting *PfLDH* [36, 43]. Metabolomics, particularly through GC–MS profiling, has enabled the identification of bioactive compounds and their metabolic impacts. Deligianni et al. [15] analysed 239 components from *Euphorbia hirta* EOs, using GC–MS data to inform ML models (KNN, LR, DT) that predicted bioactivity with an MCC of 0.701–0.840, highlighting compounds like eugenol and limonene. Although transcriptomics data are less prevalent in the provided studies, the potential for single‐cell RNA‐seq to reveal stage‐specific gene expression as observed in gametocyte‐specific *Pfg27* [23] could complement docking and ML predictions, enhancing target specificity.

Network pharmacology has further advanced target prioritisation by mapping protein–protein interactions (PPIs) and enriched pathways, integrating docking and omics data to identify functional partners and therapeutic targets. Rehman [42] employed network pharmacology to demonstrate artemisinin derivatives’ inhibition of *PfDHODH* (1TV5), with enriched KEGG pathways (pyrimidine metabolism, map00240) validated by isothermal titration calorimetry (ITC). Similarly, Mianda et al. [77] integrated UPLC–QTOF–MS, molecular docking and STRING DB‐based PPI networks to identify *PfDHODH* functional partners (*PfOPRT*, PF14_0697), with enriched GO terms like pyrimidine nucleobase biosynthesis (GO:0019856) supporting *PfDHODH* as a priority target. Elmaidomy et al. [57] combined docking, MDs and cytoscape‐based PPI networks to prioritise *20S proteasome* (7LXU) and c*holine kinase* (6YXT), with compounds like limonin and luteolin achieving ΔG‐binding < −7 kcal/mol, validated by in vitro Malstat assays. These studies illustrate how network pharmacology bridges molecular and systems‐level insights, enhancing the identification of multitarget strategies.

ML complements multiomics and network pharmacology by predicting bioactivity and refining target prioritisation. Miao et al. [14] utilised Chemprop and MAIP models with SMILES and Morgan fingerprints from *Morus alba* extracts, achieving an AUC of 0.868590 ± 0.007887 for antimalarial activity, identifying flavonoids like quercetin as key inhibitors of *PfDHFR–TS*. Deligianni et al. [15] applied KNN and LR models to GC–MS‐derived descriptors, using consensus WFI to prioritise compounds disrupting gametocyte‐to‐ookinete development. These ML models integrate omics data with docking results, enabling high‐throughput screening of phytochemicals and synthetic derivatives. For example, Oyebamiji et al. [72] used GFA with 2D descriptors (ALogP2, ATS1m) to predict binding affinities for *C. myxa* compounds, achieving an *R* ^2^ of 0.806, validated by cross‐validated QSAR models.

The strength of integrating multiomics and network pharmacology lies in its ability to provide a holistic view of *Plasmodium* biology, enabling the identification of novel targets like *PfDHODH*, *Plasmepsin II* and *Falcipain-2* through synergistic computational and experimental approaches. Robust validations, including in vitro assays (Malstat, SYBR Green I), in vivo studies (parasitaemia suppression) [42] and in silico metrics (RMSD < 2.0 Å) [53], enhance the reliability of these findings. However, limitations include challenges in data integration due to heterogeneous omics datasets, such as variable GC–MS profiles [71] or limited transcriptomic data for *Plasmodium*. Computational complexity and model interpretability, particularly in ML‐driven PPI networks, pose additional hurdles, as seen in the need for careful WFI validation [15]. The reliance on homology models (*PfCDPK-1*) [19] introduces potential inaccuracies in target prediction.

Collectively, the combination of molecular docking, gene expression profiling and ML is a multidimensional framework for antimalarial lead discovery. Docking prefers phytochemicals and synthetic compounds that have favourable binding to essential *Plasmodium* targets but is limited in itself by inaccuracies in its scoring functions and by homology model uncertainty and poor correlation to in vitro potency. Gene expression studies provide a deeper mechanistic insight, showing not only pathway modulation on target but also different compensatory responses (e.g., upregulation of glycolytic enzymes or stress pathways), as well as host–parasite interaction; however, heterogeneous experimental designs, small sample sizes and variable extract composition typically occur. ML plays a role in scalability and predictive power, for example, virtual screening, QSAR modelling and resistance prediction using large descriptor/omics‐based datasets; however, model interpretability, overfitting and reliance on biased or sparse training data are major constraints. When combined, these methods are not additive, that is, docking‐guided ML models to refine chemical space and improve the hit rates and transcriptomic readouts and pathway enrichment analyses to validate the hypotheses derived from the docking and to inform the selection of ML features, to link the predicted binding to biologically relevant phenotypes. At the same time, divergences between docking scores, ML predictions and experimental gene expression or in vitro efficacy expose context‐specific limitations and highlight where target choice, protein structure selection or training data need refinement. From a translational perspective, studies that couple in silico prioritisation with in vitro, in vivo and cytotoxicity assays offer the most credible leads, yet such fully integrated pipelines remain relatively rare and are unevenly standardised across laboratories. Future work should therefore emphasise prospective, pipeline‐based designs that intentionally combine docking, ML and multiomics readouts under harmonised protocols to generate robust, mechanism‐anchored antimalarial candidates suitable for preclinical development.

### 4.2. Discovering Antimalarial Leads via Integrated Approaches

The studies reviewed demonstrate a robust pipeline for discovering novel antimalarial leads by combining computational tools, such as molecular docking and ML, with experimental validation, including in vitro and in vivo assays. Molecular docking has been pivotal in identifying compounds with high binding affinities to critical *Plasmodium* targets, such as *PfLDH*, *PfDHODH*, *Plasmepsin II* and *PfEMP-1*. For instance, Apeh et al. [46] reported that friedelin exhibited a binding affinity of −10.6 kcal/mol to *PfDHFR*, outperforming artesunate, with in vivo validation showing significant parasitaemia reduction at 400 mg/kg. Similarly, Shah et al. [65] identified isorhamnetin as a potent *Plasmepsin II* inhibitor with an exceptionally high binding affinity of −156.33 kcal/mol, supported by favourable absorption, distribution, metabolism, excretion and toxicity (ADMET) profiles and compliance with Lipinski’s Rule of Five. These findings underscore the power of docking to prioritise compounds like 11‐O‐galloylbergenin (−16.22 kcal/mol, *PfLDH*) [23] and epicatechin‐gallate (−9.3 kcal/mol, *PfCDPK-1*) [19], which demonstrated stability in MDs simulations (RMSD < 0.4 nm) and strong in vitro antiplasmodial activity (IC_50_ < 8 µM). The integration of docking with MD simulations, as seen by Chaurasia and Pandey [48] for artocarpin (−9.6 kcal/mol, *Falcipain-2*), ensures reliable prediction of binding stability, enhancing the confidence in lead selection.

Experimental validation by in vitro and in vivo experimentation has played a huge role in proving the antiplasmodial activity of computationally found lead strategies, fortifying the relationship between in silico calculations and drug potential. Studies such as Elmaidomy et al. [57] were performed that validated Limonin’s activity against the 20S proteasome (IC_50_ = 0.2 μM) based on stable MD simulations and Malstat assays associated with ursolic acid (binding energy of the molecular complex: −9.7 kcal/mol), which correlated with 83.4% growth inhibition in vitro [45], comparable to artesunate. In vivo studies also supported these results; Camara et al. [44], for instance, reported that *T. albida* extract at 100 mg/kg completely suppressed parasitaemia by Day 4–6 and was anti‐inflammatory with a reduction in *TNF* and *IL-6* expression. Similarly, Alebachew et al. [68] revealed the ability of Knipholone (200 mg/kg) to suppress parasitaemia by 60.16% in *P. berghei*‐infected mice, with docking being able to confirm strong *PfLDH* binding (−6.96 kcal/mol). These results have demonstrated the necessity of experimental assays in corroborating computational predictions, especially in the case of compounds affecting glycolysis (*PfLDH*) [27] and hemozoin formation (*Falcipain-2/3*) [48], which play a vital role in parasite survival.

ADMET profiling and drug‐likeness calculations have also improved the lead compound selection to guarantee the potential of these to be brought into clinical development. Most of the studies reported a good oral bioavailability in accordance with Lipinski’s Rule of Five, as was seen with rhaponticin (−9.3 kcal/mol, *PfDHODH*) [54] and sesamin (−10.0 kcal/mol, *PfDHODH*) [50]. However, problems such as low Caco‐2 permeability (epicatechin‐gallate) [19] and possible toxicity (purfalcamine’s hepatotoxicity) [19] indicate the need for structural optimisation. ML has been used in complementing and going hand in hand with these efforts in order to predict bioactivity and prioritise compounds, as Miao et al. [14] showed Cosmosiin’s binding to IL6 (−7.7 kcal/mol) was validated by ML models with high AUC scores. In addition, network pharmacology approaches, such as Rehman [42], identified *PfDHODH* as an important target for artemisinin stereoisomer C6 (−8.7 kcal/mol) with validation of ITC binding specificity. These integrative approaches have improved the strength of lead identification by addressing in advance the limitations in discovery due to the pharmacokinetic and toxicological aspects.

Despite these advancements, limitations in the current studies need to be considered for future studies. Variability in docking tools (AutoDock Vina vs. Glide) and binding affinity metric (MM–PBSA) [59] can lead to difficulties when trying to directly compare between studies, and therefore, standardised protocols are needed. Additionally, while in vitro IC_50_ and inhibition curve data (aloesaponarin I, IC_50_ = 1.6 micrograms/mL) [77] as well as in vivo plasmodia suppression (*T. albida*) [44] are promising, in the absence of clinical trial data, the translation of these is limited. Compounds such as myricetin 3‐O‐glucoside (−13.413 kcal/mol, *PfLDH*) [36] offer high potential for resistance validation against resistant *Plasmodium* strains. Future research should include all types of multiomics data, like proteomics and metabolomics, to understand the mechanism of actions, such as in Chaijaroenkul et al. [70], where the upregulation of glycolytic enzymes was associated with *PfLDH* inhibition. Moreover, the extension of ML models to predict resistance profiles and the combination of single‐cell transcriptomics could be used to increase target specificity, especially for gametocyte‐specific proteins, such as *Pfg27* [23]. By helping to fill in these gaps, the integrated computational–experimental pipeline can accelerate the development of new types of antimalarial treatments that can be less prone to resistance.

### 4.3. Future Directions

Future research should prioritise in vivo validation and clinical trials to confirm the efficacy and safety of promising compounds like 11‐O‐galloylbergenin, isorhamnetin and rheediachromenoxanthone, which lack in vivo data despite potent in vitro activity [21, 43, 65]. Mechanistic studies, including knockout assays and enzyme inhibition assays, are needed to validate docking predictions (e.g., *PfLDH* inhibition by quercetin, *PfHsp70-1* disruption by betulinic acid) and elucidate molecular targets [40, 76]. Synergy testing of compound mixtures, such as EOs or plant extracts, could enhance efficacy and overcome resistance, particularly for transmission‐blocking agents like α‐pinene [7, 15]. ML models require larger, standardised datasets to improve specificity and predict resistance mechanisms, building on studies like Viira et al. [41]. Structural optimisation of compounds with high molecular weight or toxicity risks, for example, dimeric NIQs and pachyrrhizin, is critical to improve pharmacokinetics and selectivity [61, 85]. Additionally, exploring non‐oral delivery routes such as vapour sprays for EOs and multitarget strategies could broaden therapeutic applications [24, 34, 57].

### 4.4. Limitations

The reviewed studies are primarily limited by their reliance on in silico and in vitro data, with minimal in vivo validation, restricting translation to clinical settings [45, 65, 85]. Variability in docking methodologies, such as AutoDock versus MM–PBSA, and the absence of standard deviation or IC_50_ data for many compounds, hinder direct comparisons or a meta‐analysis [65, 66]. Another limitation of the docking literature is that many studies rely on a single *P. falciparum* structure as a template for other *Plasmodium* species, without systematically exploring species‐specific structural differences; although core catalytic residues are generally conserved, future work should explicitly assess orthologue conservation and, where possible, use species‐specific models. ML models suffer from low specificity due to heterogeneous training data and sparse mechanistic insights, as seen in curcuminoid predictions [41]. Most studies tested single *P. falciparum* strains (e.g., 3D7, K1), limiting insights into activity against resistant isolates [27, 40]. Toxicity concerns, such as carcinogenicity risks for compounds like afzelin and hepatotoxicity for pachyrrhizin, remain unaddressed experimentally [61, 65]. Finally, the lack of detailed pharmacokinetic profiles and synergistic effect studies for plant extracts limits their practical application [44, 67].

## 5. Conclusion

This review shows that molecular docking, gene expression profiling and ML are no longer standalone techniques but interlocking components of a coherent antimalarial discovery pipeline. Together, they have shifted the field from empirical screening towards mechanism‐informed, data‐driven prioritisation of leads from both herbal and synthetic sources. By linking target structure, pathway perturbation and predictive modelling, these approaches enable a more rational selection of compounds with genuine potential to overcome emerging resistance.

**Nomenclature**
ADMET: Absorption, distribution, metabolism, excretion, toxicity (used in drug‐likeness prediction)
MM/GBSA: Molecular mechanics/generalised Born surface area (binding energy estimation)
PfLDH: *Plasmodium falciparum* lactate dehydrogenase (drug target in glycolysis)
Plasmepsin II: Aspartic protease involved in haemoglobin degradation
pLDH: Parasite lactate dehydrogenase (antimalarial assay target)
HRP2 ELISA: Histidine‐rich protein 2 enzyme‐linked immunosorbent assay (used in malaria diagnostics)
ECM: Experimental cerebral malaria (murine model)
RMCBS: Rapid Murine Coma and Behaviour Scale (neurological scoring system for ECM)
PK/PD: Pharmacokinetics/pharmacodynamics (used for dose–response and drug action relationship)
PASS: Prediction of Activity Spectra for Substances (bioactivity prediction tool)
GMQE: Global model quality estimate (used in protein homology modelling)
UPLC–QTOF–MS: Ultra‐performance liquid chromatography–quadrupole time‐of‐flight mass spectrometry
STRING DB: Search Tool for the Retrieval of Interacting Genes/Proteins (for protein–protein interaction networks)
Pfg27: Gametocyte‐specific gene used as a transmission‐blocking marker
PfDHODH: *Plasmodium falciparum* dihydroorotate dehydrogenase (pyrimidine metabolism target)
PfCDPK‐1: *Plasmodium falciparum* calcium‐dependent protein kinase 1 (involved in erythrocyte invasion)
PfHsp70‐1/ PfHsp70‐z: Heat shock proteins involved in protein folding and parasite stress responses
Falcipain‐2/3: Cysteine proteases essential for haemoglobin digestion
PfDHFR–TS: Dihydrofolate reductase–thymidylate synthase (folate metabolism target)
PfPMT: *Plasmodium falciparum* phosphoethanolamine methyltransferase (lipid biosynthesis)
PfHGXPRT: Hypoxanthine‐guaninexanthine phosphoribosyltransferase (purine salvage pathway)
PfATP6: Sarco/endoplasmic reticulum Ca^2+^‐ATPase (targeted by artemisinin)
FAS‐II: Fatty acid synthesis pathway II (apicoplast‐localised)
PfcPRS: Cytoplasmic prolyl‐tRNA synthetase (translation machinery target)
AgEPRS: *Anopheles gambiae* glutamyl‐tRNA synthetase (vector protein synthesis target)
mJHBP: Mosquito juvenile hormone‐binding protein (vector‐based transmission target)

## Author Contributions

Reuben Samson Dangana: writing–review and editing, writing–original draft, methodology, investigation, formal analysis, data curation. Israel Ehizuelen Ebhohimen: writing–review and editing, conceptualisation. Samson Anjikwi Malgwi: writing–review and editing, proofreading. Samuel Chima Ugbaja: Moses Okpeku: writing–review and editing, proofreading, review and editing, supervision, resources, funding acquisition.

## Funding

This review did not receive any specific grant from funding agencies in the public, commercial or not‐for‐profit sectors.

## Conflicts of Interest

The authors declare no conflicts of interest.

## Data Availability

The data that support the findings of this study are available on request from the corresponding author. The data are not publicly available due to privacy or ethical restrictions.
